# The powdery mildew resistance gene *REN1 *co-segregates with an NBS-LRR gene cluster in two Central Asian grapevines

**DOI:** 10.1186/1471-2156-10-89

**Published:** 2009-12-30

**Authors:** Courtney Coleman, Dario Copetti, Guido Cipriani, Sarolta Hoffmann, Pál Kozma, László Kovács, Michele Morgante, Raffaele Testolin, Gabriele Di Gaspero

**Affiliations:** 1 Dipartimento di Scienze Agrarie e Ambientali, University of Udine, via delle Scienze 208, 33100 Udine, Italy; 2 Departments of Agriculture and Biology, Missouri State University, Springfield, MO 65897, USA; 3 Istituto di Genomica Applicata, Parco Scientifico e Tecnologico Luigi Danieli, via Jacopo Linussio 51, 33100 Udine, Italy; 4 University of Pécs, Research Institute of Viticulture and Enology, Pázmány Péter u. 4, 7634 Pécs, Hungary

## Abstract

**Background:**

Grape powdery mildew is caused by the North American native pathogen *Erysiphe necator*. Eurasian *Vitis vinifera *varieties were all believed to be susceptible. *REN1 *is the first resistance gene naturally found in cultivated plants of *Vitis vinifera*.

**Results:**

*REN1 *is present in 'Kishmish vatkana' and 'Dzhandzhal kara', two grapevines documented in Central Asia since the 1920's. These cultivars have a second-degree relationship (half sibs, grandparent-grandchild, or avuncular), and share by descent the chromosome on which the resistance allele *REN1 *is located. The *REN1 *interval was restricted to 1.4 cM using 38 SSR markers distributed across the locus and the segregation of the resistance phenotype in two progenies of collectively 461 offspring, derived from either resistant parent. The boundary markers delimit a 1.4-Mbp sequence in the PN40024 reference genome, which contains 27 genes with known functions, 2 full-length coiled-coil NBS-LRR genes, and 9 NBS-LRR pseudogenes. In the *REN1 *locus of PN40024, NBS genes have proliferated through a mixture of segmental duplications, tandem gene duplications, and intragenic recombination between paralogues, indicating that the *REN1 *locus has been inherently prone to producing genetic variation. Three SSR markers co-segregate with *REN1*, the outer ones confining the 908-kb array of NBS-LRR genes. Kinship and clustering analyses based on genetic distances with susceptible cultivars representative of Central Asian *Vitis vinifera *indicated that 'Kishmish vatkana' and 'Dzhandzhal kara' fit well into local germplasm. 'Kishmish vatkana' also has a parent-offspring relationship with the seedless table grape 'Sultanina'. In addition, the distant genetic relatedness to rootstocks, some of which are derived from North American species resistant to powdery mildew and have been used worldwide to guard against phylloxera since the late 1800's, argues against *REN1 *being infused into *Vitis vinifera *from a recent interspecific hybridisation.

**Conclusion:**

The *REN1 *gene resides in an NBS-LRR gene cluster tightly delimited by two flanking SSR markers, which can assist in the selection of this DNA block in breeding between *Vitis vinifera *cultivars. The *REN1 *locus has multiple layers of structural complexity compared with its two closely related paralogous NBS clusters, which are located some 5 Mbp upstream and 4 Mbp downstream of the *REN1 *interval on the same chromosome.

## Background

The repertoire of grapevine resistance genes against fungal diseases has recently embraced a novel member, the first naturally present in a genome of the cultivated species *Vitis vinifera *[1]. Grapevines were domesticated in the Mediterranean basin, in Western and Central Asia, and then cultivated in all temperate regions around the world [2,3]. Western Eurasian cultivars all succumb to powdery mildew outbreaks in unprotected vineyards, following the worldwide spread of the pathogen from the American continent since the second half of the 19^th ^century [4]. Russian breeders sought after sources of resistance in Central Asian varieties, which kindled hopes of finding resistance genes in cultivated vines (reviewed in [5]). 'Kishmish vatkana' is a seedless grape originally found at the beginning of the 20^th ^century in the Uzbek oasis of Shakhrisabz, about 70 km south of Samarqand, and then maintained in local germplasm collections. 'Kishmish vatkana' is able to mount a post-penetration reaction against *Erysiphe necator *Schwein, a trait controlled by the dominant gene *REN1 *[1]. *REN1 *is a novel resistance gene located on chromosome 13, different from those that had been previously found in wild North American grapes and their interspecific hybrids [6-12].

*REN1 *or other resistance genes might be present in other accessions of *V. vinifera *grown in Armenia, Moldova, Russia, Georgia, and Uzbekistan (reviewed in [5]). Another cultivar called 'Dzhandzhal kara', also written as 'Djandjal Kara', "Karadzhandal', and 'Janjal kara' in Western literature [13,14] and identified with the synonym 'Semiz kara', has been utilised by breeders as a source of powdery mildew resistance [14,15]. 'Dzhandzhal kara' was first reported in the Uzbek district of Urgut, about 40 km southeast of Samarqand, and a few more stocks also existed in Kitab, within a 10-km range from Shakhrisabz where 'Kishmish vatkana' was also found. 'Dzhandzhal kara' is cultivated for the production of table grapes. 'Dzhandzhal kara' has normal hermaphrodite flowers that develop viable embryos and yield seeded fruit. 'Kishmish vatkana' is regarded as a stenospermocarpic cultivar, because its ovules degenerate soon after fertilisation at the initial stages of embryogenesis. 'Kishmish vatkana' and 'Dzhandzhal kara' survive natural powdery mildew infections better than any *V. vinifera *cultivar. Under high disease pressure, *E. necator *occasionally escapes the plant's defence reaction, which leads to microcolony formation and limited conidiation in both cultivars, with 'Dzhandzhal kara' allowing slightly more hyphal growth than 'Kishmish vatkana' (P. Kozma, unpublished results). Adaptive evolution of *V. vinifera *under the selective pressure of the pathogen was generally disregarded, because this crop is vegetatively propagated, and plants in the wild have probably undergone few mating generations since the arrival of the pathogen to the Eurasian continent. Thus, one might suspect a recent hybridisation with a resistant non-*V. vinifera *ancestor accidentally present in the area of cultivation, such as a North American rootstock or a wild grape from East Asia brought to the fertile oases of Central Asia along the ancient trade routes to the west [16]. 'Kishmish vatkana' and 'Dzhandzhal kara' are unique within the species *V. vinifera *in their resistance to powdery mildew, but they are typical among domesticated varieties in all other respects, particularly when compared with varieties grown in the Uzbek oases where 'Kishmish vatkana' and 'Dzhandzhal kara' were found, such as 'Nimrang' and 'Katta kurgan'. 'Nimrang' and 'Katta kurgan' have been imported, utilised, and documented in Western and in Far Eastern literature since the middle of the last century [17-21]. Their pure *V. vinifera *origin has never been called into question: instead they are credited with some of the most distinguishing features of the *V. vinifera *subsp. *sativa *group orientalis subgroup antasiatica [22,23]. Similarly, 'Kishmish vatkana' and 'Dzhandzhal kara' conform to many morphological descriptors that differentiate *V. vinifera *varieties from wild relatives, and thus do not appear to be the result of a recent genome introgression from feral grapevines.

If the *REN1 *gene had not moved into the *V. vinifera *germplasm from outside, the questions remain as to which functional class this gene belongs to and how it has evolved into functionality. Histological analyses have shown that *REN1 *does not prevent cell entry in 'Kishmish vatkana' but it inhibits the progression of pathogen invasion once haustoria have penetrated the host cells [1]. This mode of action bears a strong resemblance to that of host resistance genes. Many plant resistances to powdery mildews are controlled by NBS-LRR genes, which all confer post penetration resistance [24], including *Run1 *in the muscadine grape [9]. NBS-LRR genes have proliferated through reiterate tandem duplication in plant genomes, forming gene arrays prone to random shuffling of DNA segments. Genes in this class evolve rapidly, with their clustered organisation facilitating some mutated members to express novel receptors that may match the effectors of a virulent pathogen. In vegetatively propagated crops, *R*-genes are more likely to develop in wild populations or at the outer margins of the field crops, where plants sexually propagate and generate genetic novelty, some of which might provide a competitive advantage.

In this paper, we show (1) the presence of *REN1 *in a second Central Asian variety named 'Dzhandzhal kara', which has some distinctive viticultural traits with respect to 'Kishmish vatkana' (e.g. seeded vs. seedless table grape); (2) the precise location of the *REN1 *locus using recombinants from 461 offspring in two separate groups of progeny, one from each resistant variety crossed with susceptible grapevines; (3) gene content, structural organisation, and dynamics of evolution of the homologous 1.4-Mb *REN1 *region in the sequenced grapevine PN40024; (4) kinship and population analyses for uncovering the genetic background of 'Kishmish vatkana' and 'Dzhandzhal kara'.

## Methods

### Plant material and evaluation of powdery mildew resistance

'Kishmish vatkana' and the progeny derived from its cross with 'Nimrang' (04-10/nnn, where nnn is the identifier of each seedling) are described in [1]. 'Dzhandzhal kara' was kindly provided by P. Cindric', University of Novi Sad, Serbia. 'Dzhandzhal kara' is one of the grandparents of the segregating population derived from the cross ('Dzhandzhal kara' × 'Lasta') × ('Katta kurgan' × 'Perlette'), coded 07-12/nnn. Lasta is a downy mildew resistant descendent of 'Villard blanc'. The 'Dzhandzhal kara' × 'Lasta' offspring named SK91-4/27, later used for further crossing, was selected at the University of Novi Sad, Serbia, with the aim of combining downy and powdery mildew resistances with table grape traits. The other grandparents are the table varieties 'Katta kurgan' and 'Perlette', a traditional Central Asian grapevine and a recently bred *V. vinifera *intraspecific hybrid, respectively. Except for 'Dzhandzhal kara', all grandparents are susceptible to powdery mildew, and 'Villard blanc' is the only non-*V. vinifera *ancestor in the pedigree of the 07-12 population. All grandparents were analysed in order to trace the origin of each allele segregating in the progeny. The 07-12 progeny were evaluated for powdery mildew resistance using the same procedure previously adopted for the 04-10 progeny [1].

### Development and mapping of markers in the *REN1 *region

A search for microsatellites around the *REN1 *region was performed within the homologous interval of the PN40024 genome sequence using a modified version of the Sputnik repeat-finder [25,26]. SSR primers were designed using Primer 3. The uniqueness of their annealing sites was confirmed by a BlastN search of the primer sequences against the PN40024 genome sequence. Genomic DNA was extracted from young leaves. Segregation analysis was performed as reported in [1]. Local linkage maps were constructed using CarthaGene [27].

### Gene annotation and structural analyses of the *REN1 *locus

Gene models and repetitive elements were predicted using GAZE, Repeat Masker, Tandem Repeats Finder, RepeatScout, and ReAS [28]. GAZE gene models were annotated by a BlastX search of the NCBI protein database, and assigned to the predicted function of their best match. The alignment of RNA-Seq short reads to the genomic DNA sequence was used to find further putative coding regions outside of the GAZE models [29].

A representative set of NBS-LRR proteins from *Arabidopsis *and rice [30,31] was used for a tBLASTn search of the *REN1 *region in order to uncover gene fragments and pseudogenes. The matching regions and the corresponding NBS models were numbered progressively based on their genome position. Each sequence displaying similarity supported by an E value of < e^-10 ^was extended on each side until the next predicted gene, and manually annotated using a gene prediction consensus obtained with GenScan, FGENESH, GENEMARK, and Geneid. Amino acid alignments to other plant CC-NBS-LRR proteins guided the choice of the most likely gene model when gene finders provided conflicting predictions. The deduced amino acid sequences were aligned to reference plant CC-NBS-LRR proteins using the module Blast 2 Sequences. Protein motifs were predicted using Pfam, SMART, and COILS. Paralogous NBS genes of the NBS members found in the *REN1 *interval were identified by the best reciprocal match, and retained at the thresholds of E values < e^-10 ^and significant identity over > 100 bp. Amino acid alignments were carried out using ClustalX. Similarity trees were constructed using the Neighbor Joining (NJ) algorithm implemented in MEGA, with 1000 bootstrap replicates. Comparison of duplicated regions and drawing of DNA dot plots were performed using Dotter. Global DNA alignments were done using LAGAN in a window of 100 bp with a minimum identity of 70%, and visualised with VISTA. Nucleotide positions along chr13 refer to the version of the grapevine genome assembly deposited under the accession number FN597036.

### Kinship and population analyses

Genotypic data were produced using a set of 24 SSR markers, covering all chromosomes and including a standard set of 6 markers for homogenisation with the European Vitis Database [32]. Genomic DNA was PCR-amplified using 200 μM of each dNTP, 1 U of *Taq *polymerase, and 2.5 pmol of each primer at an annealing temperature of 55°C with 30 PCR cycles. PCR products were run on a Beckman Coulter CEQ 8000 capillary sequencer and alleles were sized with the CEQ 8000 Genetic Analysis software, Fragment Analysis Module.

Kinship analysis was performed using KINGROUP v2 [33]. Allele frequencies at 19 unlinked SSR loci (one per chromosome) were calculated from a sample of 16 cultivars representative of the gene pool of *V. vinifera *ssp. *sativa *convarietas orientalis and pontica, which include grapes from Central Asia and from regions around the Black Sea. Pairwise relatedness (*r*), which is a measure of the proportion of alleles in common that are identical by descent, and associated *p*-values were estimated for each possible pair of cultivars using the algorithm from [34]. Then, a likelihood ratio (LR), with associated *p*-values, was calculated for each possible relationship category between the pairs, using a hypothesized relationship as the primary hypothesis, and the next closest relationship category as the null hypothesis.

Clustering was performed using NTSYS. For the 16 accessions used in the kinship analysis, genetic similarity was calculated using a simple match coefficient and clustering was performed with the unweighted pair-group method with arithmetic mean (UPGMA) and NJ algorithms. For the extended germplasm comparison, genotypic data at six markers recommended by the European Vitis Database were obtained from public databases [35,36] and literature. Similarity was calculated based on a distance coefficient (DIST) in order to handle the lack of harmonisation of allelic sizes from different databases. Principal component analysis (PCA) was based on standardised covariance of genetic distances between 'Kishmish vatkana', 'Dzhandzhal kara', and alleles of 216 other accessions. PCA was performed using GenAlEx [37].

## Results

### Genetic determinant of PM resistance in 'Dzhandzhal kara'

Powdery mildew resistance is transmitted by 'Dzhandzhal kara' as a Mendelian trait. The inheritance of PM resistance was investigated using an available cross population, hereafter referred to as the 07-12 population, where 'Dzhandzhal kara' is one of the grandparents, the parents being ('Dzhandzhal kara' × 'Lasta') × ('Katta kurgan' × 'Perlette'). Each of the grandparents is heterogeneously heterozygous and homozygous at a genome-wide level, while they are *Rr*, *rr*, *rr*, and *rr *for the PM resistance phenotypic trait, respectively. The parents of the 07-12 population are *Rr *and *rr*, respectively. Thus, the 07-12 segregating population is formally an intercross or cross population, according to the nomenclature given by popular mapping software such as Carthagene and JoinMap. However, this kind of population is the best approximation for a classical testcross in the genetic analysis of a heterozygous *Rr *trait in outcrossing species, in the absence of full homozygous lines.

The inheritance of PM resistance was studied in 151 offspring of the 07-12 population by scoring the presence or absence of visible symptoms. Of these, 71 siblings showed masses of conidiophores detectable to the naked eye, while 80 siblings showed no symptoms during the entire vegetative season. The observed segregation ratio fits a monogenic control of the trait (1:1, χ^2 ^= 0.12), as previously observed for 'Kishmish vatkana' [1].

In order to prove that (i) the parent 'Dzhandzhal kara' × 'Lasta' is heterozygous for the resistance allele that controls this trait, (ii) the resistance allele was inherited from the grandparent 'Dzhandzhal kara', and (iii) to assess if this allele is the same as in 'Kishmish vatkana', a test of independence was performed in the 07-12 progeny with markers linked to *REN1 *and the alleles were traced back to the parents and grandparents. The SSR markers UDV124, VMC9H4-2, and VMCNG4E10-1 that flank *REN1 *[1], and three markers that co-segregate with the resistance locus in 'Kishmish vatkana' (see below) were used for this screening. Out of these markers, UDV124 was the only homozygous marker in 'Dzhandzhal kara' × 'Lasta' and it was substituted with the heterozygous marker sc147_2, which is located 180 kb away from UDV124 on the PN40024 reference sequence. One allele at each of these loci was found to be shared by 'Kishmish vatkana', 'Dzhandzhal kara' × 'Lasta', and 'Dzhandzhal kara', and the same allele was associated with the resistant half of the seedlings bred from 'Dzhandzhal kara' × 'Lasta' (Table 1). Thus, the same SSR alleles in coupling with powdery mildew resistance in 'Kishmish vatkana' are present only in 'Dzhandzhal kara' among the four grandparents of the 07-12 family, and are inherited only by the resistant descendents, except for the few cases in which a recombination between the markers and the *REN1 *gene has occurred.

**Table 1:** Test of independence for six markers, found to be linked to *REN1 *in 'Kishmish vatkana', and phenotypes of powdery mildew resistance/susceptibility in a ('Dzhandzhal kara' × 'Lasta') × ('Katta kurgan' × 'Perlette') F1 family.

Marker	Resistant F1s		Susceptible F1s		χ^2 ^test of independence
		
	Allele *A*	Allele *a*	Allele *A*	Allele *a*	
sc147_2	77 (**180**)	3 (164)	2 (**180**)	69 (164)	132.5**
VMC9H4-2	79 (**283**)	1 (289)	0 (**283**)	71 (289)	147.8**
VMCNG4E10-1	79 (**259**)	1 (264)	0 (**259**)	71 (264)	147.8**
SC8_0071_014	80 (**163**)	0 (181)	0 (**163**)	71 (181)	152.1**
sc47_19	80 (**129**)	0 (null)	0 (**129**)	71 (null)	152.1**
sc47_18	80 (**268**)	0 (257)	0 (**268**)	71 (257)	152.1**

The markers sc147_2, VMC9H4-2, and VMCNG4E10-1 flank each side of *REN1 *in 'Kishmish vatkana', markers SC8_0071_014, sc47_19, and sc47_18 co-segregate with *REN1 *in 'Kishmish vatkana'. Allele size is given between parentheses in base pairs, the alleles inherited from 'Dzhandzhal kara' are bold faced. Asterisks indicate *p *> 0.01 for 3 df.

### Marker saturation of the *REN1 *interval in 'Kishmish vatkana'

**Figure 1:**
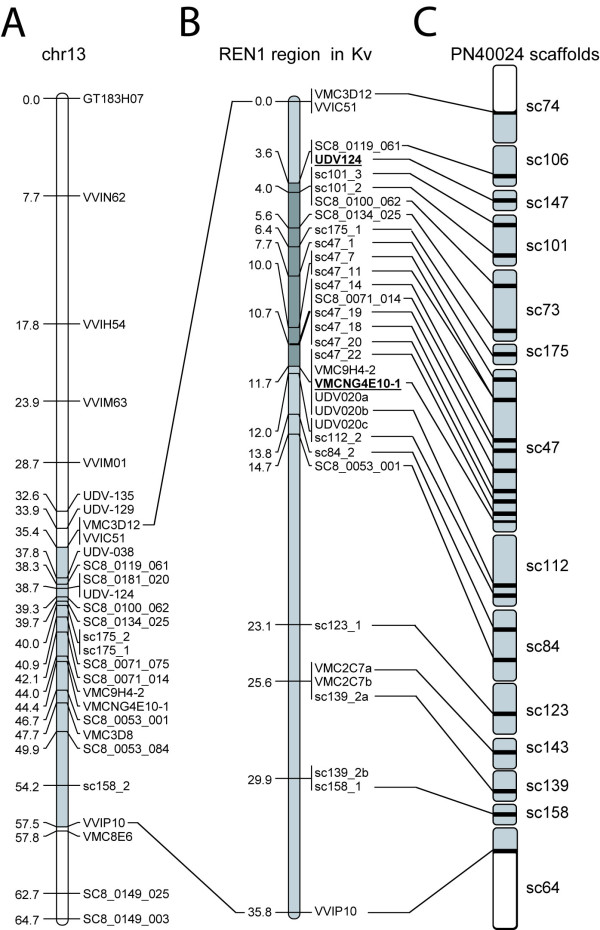
**Saturation of the *REN1 *interval in 'Kishmish vatkana' and links to the PN40024 sequence assembly**. (**A**) Reference linkage map of chr13, as reported in [38]. (**B**) Local linkage map in 'Kishmish vatkana' around the former *REN1 *interval (light grey segment) comprised between markers VMC3D12-VVIC51 and VVIP10 as previously defined by [1]. The former *REN1 *region (dark grey segment) as identified by the heterozygous markers available in [1] was delimited by markers (bold) UDV124 on the proximal side and VMC9H4-2 and VMC4E10-1 on the distal side of chr13. (**C**) Sequence scaffolds in the PN40024 genome assembly. Markers already present on the previous version of the 'Kishmish vatkana' map [1] were projected onto the scaffolds. Scaffolds whose relative positions were known in PN40024 and for which markers were lacking in the 'Kishmish vatkana' map were used for developing targeted markers that saturate the *REN1 *interval.

Markers across the *REN1*interval between VMC3d12-VVIC51 and VVIP10, previously identified on 'Kishmish vatkana' chr13 [1], were projected onto the PN40024 genome sequence by a BlastN search of their primer sequences. Other markers in the same interval were gathered from the reference genetic map used for anchoring the PN40024 genome sequence (Figure 1). This two-pronged approach enabled the identification of the genome sequence scaffolds covering the *REN1 *interval. All scaffolds lacking an anchor to the 'Kishmish vatkana' local map were mapped using scaffold-specific SSR markers selected from a reference genetic map [38]. At least one marker per scaffold was placed in the genetic map (Figure 1). New markers were developed using the reference sequence in the region between UDV124 and VMC9H4-2/VMCNG4E10-1 at regular physical intervals. The candidate interval around *REN1 *was then filled with 14 markers heterozygous in 'Kishmish vatkana'. *REN1 *was previously placed 0.9 cM proximal to VMC9H4-2/VMC4E10-1: the relative order between these markers and the locus was based on three recombinants, but the surrounding genome landscape was highly repetitive, with only multi-locus markers available [1]. Hence, outer markers were also developed on scaffolds flanking the initial interval on the distal side, in order to confirm the outer border with more recombinants and more markers. The saturated local map of 'Kishmish vatkana' is shown in Figure 1. For details of all markers [see Additional file 1]. Using the recombinations that occurred in 310 germ cells of 'Kishmish vatkana', the genetic interval was restricted to a region corresponding to a block of continuous DNA sequence in PN40024, contained in scaffold_47. Markers developed on scaffolds distal to VMC9H4-2/VMC4E10-1 provided further evidence for excluding the possibility of the resistance locus being located in the repetitive region outside of the pinpointed interval (Figure 1).

### Restriction of the *REN1 *interval based on crossovers in 'Kishmish vatkana' and 'Dzhandzhal kara' × 'Lasta'

**Figure 2:**
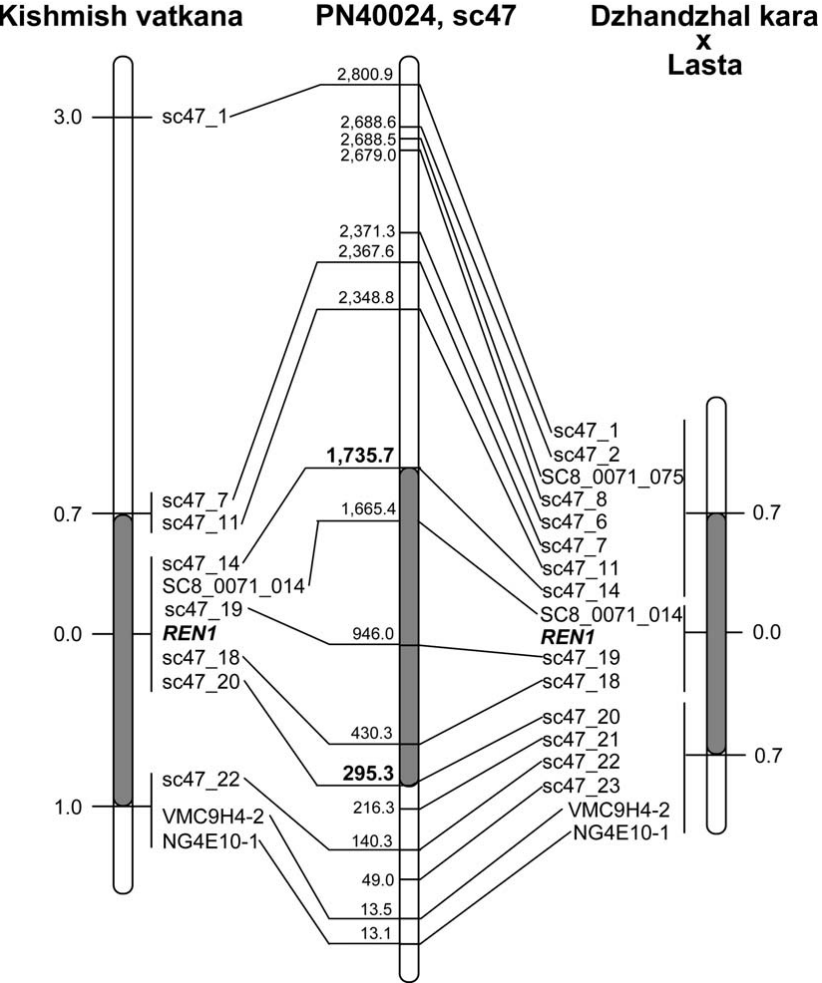
**Genetic interval of the *REN1 *region (grey segment) in the 'Kishmish vatkana' and 'Dzhandzhal kara' × 'Lasta' local maps, and projection of the related markers on the PN40024 sequence**. Distances in the genetic maps are given in cM, calculated from the *REN1 *position outwards. Distances in the sequence scaffold sc47 are given in kbp from the bottom of the scaffold, which is oriented (-) in the PN40024 sequence assembly.

SSR markers were developed along scaffold_47 at regular physical intervals [see Additional file 1], and mapped in 'Kishmish vatkana' and/or 'Dzhandzhal kara' × 'Lasta', according to their heterozygous state. Local genetic maps around the *REN1 *locus across scaffold_47 encompass 11 informative markers in 'Kishmish vatkana' and 17 informative markers in 'Dzhandzhal kara' × 'Lasta' (Figure 2). In 'Kishmish vatkana', *REN1 *co-segregated with 5 markers: sc47_14, SC8_0071_014, sc47_19, sc47_18, and sc47_20. The closest flanking markers are sc47_7 and sc47_11 which are located 0.7 cM away on the upper side of *REN1*, and sc47_22, VMC9H4-2, and VMCNG4E10-1 which are placed at 1.0 cM on the opposite side. In 'Dzhandzhal kara' × 'Lasta', *REN1 *co-segregated with 3 markers: SC8_0071_014, sc47_19, and sc47_18. The border on the upper side is defined by a group of 8 co-segregating markers 0.7 cM away from *REN1*, and the lower side of the *REN1 *interval is bordered by a symmetric group of 6 co-segregating markers at a distance of 0.7 cM. The closest flanking markers correspond to a physical distance of 1.4 Mbp in the PN40024 sequence.

The haplotypes of the recombinant individuals that progressively restricted the interval down to scaffold_47 and to the 1.4 Mbp region within scaffold_47 were visually inspected and compared with their corresponding phenotype [see Additional file 2]. Genotyping of 310 offspring of 'Kishmish vatkana' revealed four recombination events that occurred most closely to *REN1 *in the germ cells that gave rise to the individuals 04-10/48 and 04-10/248, which both have a one-point crossover between markers s47_11 and s47_14, and in the germ cells that gave rise to the individuals 04-10/3 and 04-10/337, whose chromosome (chr) 13 tetrads recombined between sc47_20 and sc47_22, on the opposite side of *REN1*. The interval between sc47_11 and sc47_22 corresponds to 1.7 cM in the 'Kishmish vatkana' genetic map, and to a physical distance of 2.2 Mbp in PN40024. Despite being sampled from a smaller family of 151 offspring, the most informative recombinants in the 'Dzhandzhal kara' × 'Lasta' descendents originated from crossovers closer to *REN1 *on both sides of the gene. The two closest recombination events occurred in the germ cells that gave rise to the offspring 07-12/82, which bore a crossover between the markers sc47_14 and SC8_0071_014 on the upper side of the gene, and the offspring 07-12/139, which bore a crossover between sc47_18 and sc47_20 downstream of the gene. The interval between markers sc47_14 and sc47_20 is 1.4-cM long in the 'Dzhandzhal kara' × 'Lasta' genetic map, and corresponds to 1.4 Mbp in the PN40024 genome assembly.

### The *REN1 *region in PN40024: genes and structural features

The *REN1 *region from marker sc47_14 to sc47_20 spans 1,440,612 bp in the homologous interval of the PN40024 genome. Within this region there are 27 genes predicted by GAZE and BlastX that putatively encompass 15 different functions, and 11 other gene models that encode full-length or partial NBS-LRR disease resistance genes with a coiled-coil domain in each N-terminus [see Additional file 3 and Additional file 4]. The hypothetical protein products of 11 other gene models had no significant match in NCBI. The average gene density is one gene every 28.8 kb. Gene distribution is quite even and complementary to the repetitive fraction, which overall covers 29% of the interval.

**Figure 3:**
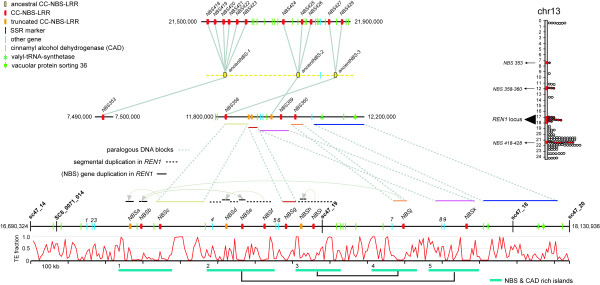
**Diagram of gene content and structural organisation of the *REN1 *interval in PN40024 (chr13:16690324..18130936, horizontal panel at the bottom), and relationships with its paralogous loci on the same chromosome (other horizontal panels)**. The distribution of dispersed NBS genes and NBS gene clusters along chr13 are shown as solid and open circles in the diagram on the right, with distances along the chromosomes given in Mbp. Blocks containing NBS genes at the *REN1 *locus and at its paralogous loci (chr13:11.8..12.2 Mb and chr13:21.5..21.9 Mb) are shown as red boxes along the vertical diagram of chr 13, and are magnified in the horizontal panels in the middle of the figure. Conserved regions between chr13:11.8..12.2 Mb and *REN1*, as assessed by dotplot comparison [see Additional file 5], and identity of conserved nucleotide sequences (>70% identity in a sliding window of 100 bp) are indicated by horizontal coloured bars. NBS and CAD gene-rich islands within the *REN1 *locus were identified by dotplot self-comparison [see Additional file 5] and are indicated at the bottom of the figure by horizontal cyan bars (1 to 5). Connections between gene-rich islands are defined based on the percentage of conserved non-coding sequence. NBS gene relationships within *REN1 *in segmentally duplicated fragments and in single-gene duplications are defined based on the percentage of conserved sequence in blocks of 10 kb around each gene (Figure 4). The fraction of transposable elements (TEs) in a sliding window of 5 kb along the *REN1 *locus is given below the locus bar. Full length (red) and partial (orange) CC-NBS-LRR genes are indicated by boxes with their orientation and named from NBSa to NBSk; complete and partial genes of other categories [see Additional file 3] are shown as green and cyan ticks; microsatellite markers are shown by black ticks. Gene models are not drawn to scale. Evolutionary dynamics that have shaped these chromosomal regions from three NBS-LRR founders (in yellow) to the present-day structure of the *REN1 *locus and its paralogous loci are described in the text.

**Figure 4:**
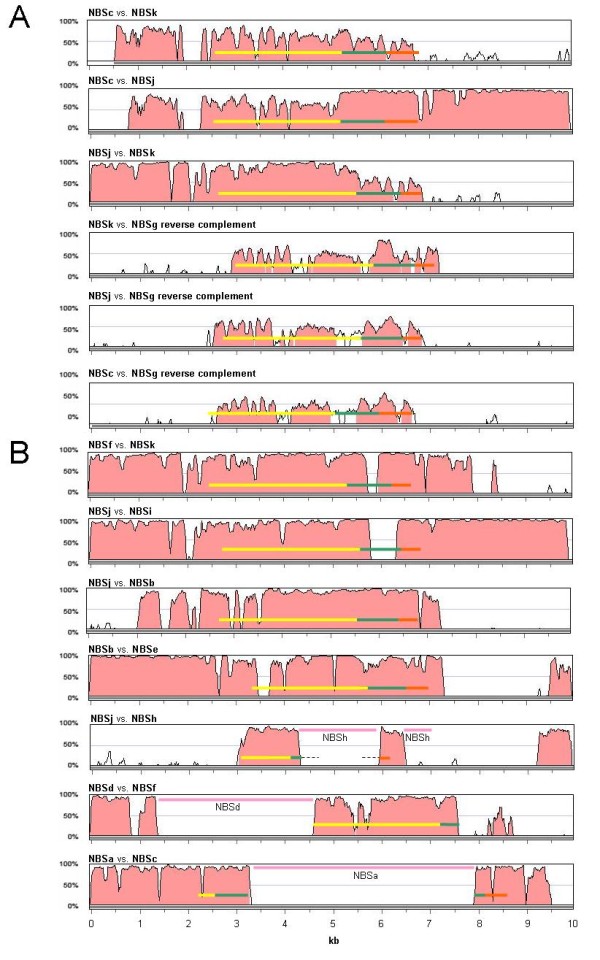
**Global alignment of 10-kb sequences around each NBS gene in the *REN1 *interval**. (**A**) Comparison between the NBS founders of the *REN1 *interval (NBSc, NBSg, NBSj, NBSk). (**B**) Comparison between NBS gene pairs that have evolved from a duplication of a common ancestral sequence within the *REN1 *interval, based on the widest sharing of the highest nucleotide identity. The conserved blocks of nucleotide sequences and the corresponding level of identity were calculated using LAGAN and drawn using VISTA. The coding regions corresponding to the coiled coil (orange), NBS (green), and LRR (yellow) domains are indicated by coloured bars. The NBS domain was defined as the portion comprised from the P-loop to the MHDV motif. The insertion of repetitive elements is indicated by pink bars with the identifier of the gene in which the insertion has occurred. DNA sequences are represented from left to right, according to the top-to-bottom orientation of the assembled genome sequence along chr13. All protein coding sequences, with the exception of NBSg, are represented in an N- to C-terminus orientation from right to left.

The 11 NBS-LRR gene models contained in the *REN1 *interval are arrayed over a physical distance of 908 kb, comprised between the markers SC8_0071_014 and sc47_18 (Figure 3). Even though chr13 is the richest chromosome in NBS genes, with more than a hundred genes and pseudogenes, no other NBS gene was found nearby in the same scaffold. The closest NBS genes outside of this interval are located 3.4 Mb upstream of *REN1 *on scaffold_73, and downstream on scaffolds_112 and 84, respectively (see Figure 1). The NBS genes in the *REN1 *interval showed the highest identity with CC-NBS-LRR resistance genes from soybean (*Rps1*-k) and potato (*R3a*), in the range of 34-38% amino acid identity averaged over the CC, NBS, and LRR domains. Two NBS genes of the region, named NBSj and NBSk, are predicted by FGENESH and GENEMARK to encode full-length gene products of 1,436 and 1,428 amino acids, respectively, while all other coding sequences are disrupted in PN40024 by small frameshift indels or insertion of transposable elements [see Additional file 4]. COILS predicted the presence of a coiled coil structure for all genes, except for NBSd, for which the N terminus is missing. The NBS domain was invariably present, and the LRR region varied in length. The level of nucleotide identity between NBS-LRR gene models and across the CC, NBS, and LRR domains, and in the 5' and 3' non-coding regions is shown in Figure 4.

As for other genes with putative functions that might be physiologically relevant to disease resistance, the most abundant gene category was represented by 9 cinnamyl alcohol dehydrogenases (CADs) involved in lignin biosynthesis, two WD40-repeat proteins, a kinase-like protein, and a Rab1/RabD small GTPase [see Additional file 3 for the complete list of genes in the region]. Among these, 6 CADs, 2 DNA gyrases, a dihydropyrimidinase, a kinase-like protein, an NADH-dehydrogenase, a vacuolar protein, and a tRNA^val ^synthetase showed evidence of constitutive expression in plantlets of PN40024, as assessed by deep RNA-Seq [29].

### NBS paralogues in the *REN1 *region: local and ectopic duplications

**Figure 5:**
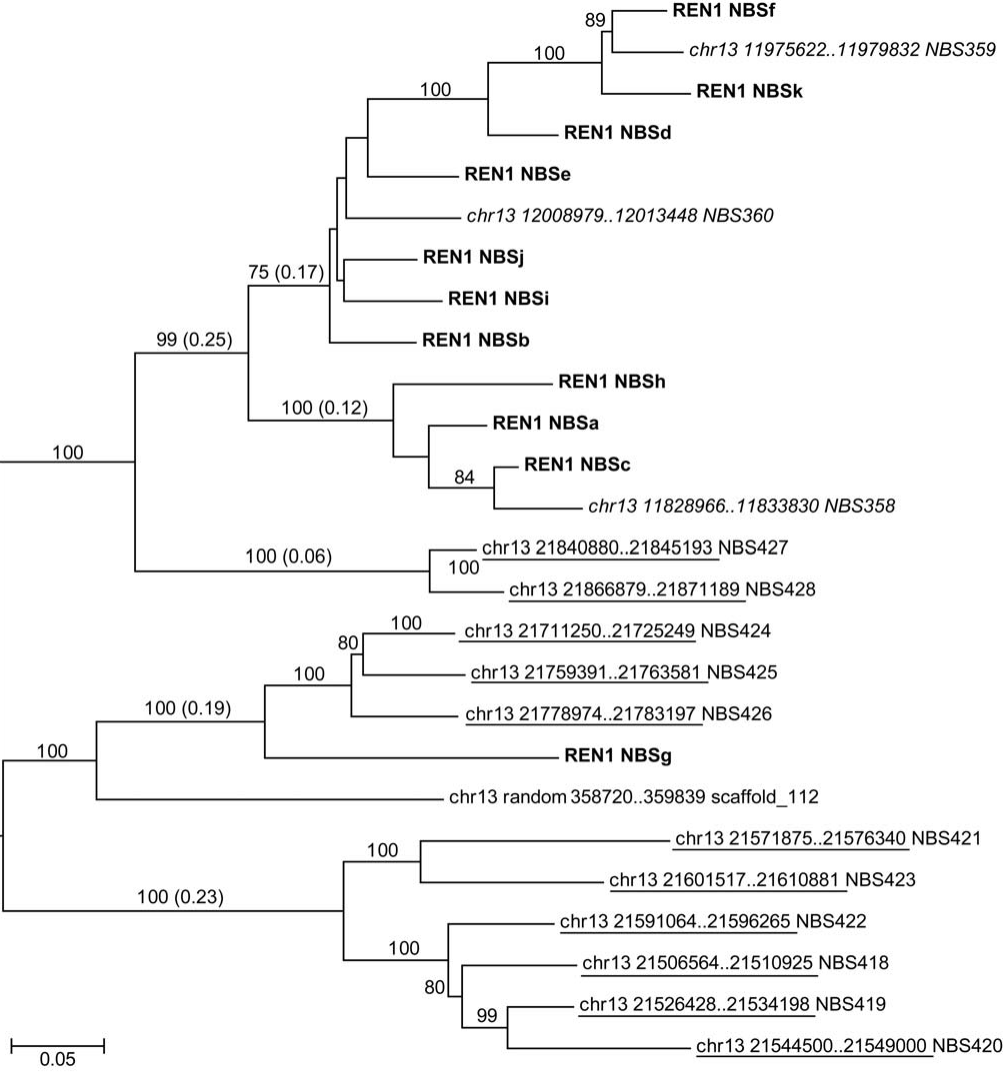
**Neighbor joining tree of 11 NBS genes in the *REN1 *interval (bold) and their most similar paralogues in the grape genome, which are 3 NBS genes in the interval chr13:11828966..12013448 (italics) and 11 NBS genes in the interval chr13:21506564..21871189 (underlined)**. The NJ coefficient is based on amino acid alignments. Bootstrapping was performed with 1000 replicates and the percentage of replicates supporting that branch is given above the branch (if >70). The estimates of average evolutionary divergence between NBS-LRR sequences within groups are indicated in parentheses above the corresponding branch. The number of amino acid substitutions per site from averaging over all sequence pairs within each group was calculated using the Poisson correction method in MEGA. All positions containing alignment gaps or missing data were eliminated only in pairwise sequence comparisons (Pairwise deletion option).

In order to shed light on the structural organisation of the *REN1 *locus, a BLAST search using the NBS genes in the *REN1 *interval as a query was performed against the whole genome of PN40024, and the best three matches were retained for each query. The best matches were most frequently found between pairwise comparisons of NBS genes within the *REN1 *locus (chr13:16.7..18.1 Mb), but in some cases also between an NBS gene of the *REN1 *locus and an NBS gene residing in either of the two other gene clusters dispersed along chr13, one composed of 3 members and located approximately 5 Mbp upstream of the *REN1 *interval (chr13:11.8..12.2 Mb) and the other composed of 11 members and located approximately 4 Mbp downstream of *REN1 *(chr13:21.5..21.9 Mb, Figure 3). The relationships between the NBS members in the *REN1 *region and in the paralogous clusters are shown in a NJ tree (Figure 5). The estimated average evolutionary divergence (*d*) over NBS pairs within the *REN1 *interval is 0.30 (*d *= 0.24 if NBSg, the most divergent one and the only one in opposite orientation with respect to all others, is omitted from the analysis), which is lower than the values found within the upper cluster (*d *= 0.35) and within the lower cluster (*d *= 0.40). NBS genes in the *REN1 *cluster had a closer relatedness with those in the upper cluster, based on the average estimated evolutionary divergence among NBS pairs between the clusters (*d *= 0.28), in comparison with the lower cluster (*d *= 0.55). The distance between NBS genes in the upper and the lower clusters was also 0.55. If the connection between sequence similarity and physical proximity between NBS pairs is disregarded, the estimated divergence within NJ clades, some including individual members that reside in different physical clusters, is far lower (Figure 5). Overall, these observations are indicative of the concomitant contribution of local and ectopic duplications in the evolution of the present-day *REN1 *locus.

The role of segmental duplications and/or small-scale gene duplication between and within gene clusters was assessed through three complementary approaches: dot plot analyses, global alignments of the entire paralogous segments (from 400 kb to 1.4 Mb), and percentage of conserved sequences in pairwise alignments of the 10-kb gene environment around each NBS-LRR gene.

There are 261.7-kb of conserved non-coding sequences in the chr13:11.8..12.2 Mb cluster (65% of the total length) that are shared with the *REN1 *interval, at an average identity of 93.9%. Physical expansion of the *REN1 *interval can be largely attributed to segmental local duplications of DNA blocks containing NBS and CAD genes and also by colonisation of genic and intergenic space by TEs (Figure 3). Overall, these events caused an approximate three-fold expansion of the *REN1 *region with respect to the paralogous locus chr13:11.8..12.2 Mb. Regions of extended colinearity are evident by dot plot comparison in the distal part of the two clusters, from immediately upstream of NBSk to the end of the *REN1 *interval, as well as at and downstream of the NBSc location [see Additional file 5]. Large duplications did not occur in the chr13:11.8..12.2 Mb cluster [see Additional file 5]. The relationship between the *REN1 *interval and the lower NBS cluster (chr13:21.5..21.9 Mb) is more limited: they share only 3.9% of non-coding sequences, but gene order and orientation of two progenitor NBS paralogues and a CAD gene in chr13:21.5..21.9 Mb are conserved, including the paralogue of NBSg, the only NBS with forward orientation in the *REN1 *interval [see Additional file 5]. Similar levels of conserved sequences (2.6%) and dot plot colinearity are recognisable between the chr13:11.8..12.2 Mb cluster and the chr13:21.5..21.9 Mb cluster [see Additional file 5].

Based on the level of sequence conservation in non-coding regions over the entirety of these intervals, and the nucleotide similarity in the 10-kb NBS gene environment between and within clusters and their best reciprocal matches, the most parsimonious model of evolution, which explains the present-day organisation of the *REN1 *locus in PN40024, is as follows. Three progenitor NBS-LRR genes, structured in a proto-cluster in an ancestral genome, generated the current genomic structure of the *REN1 *locus and its paralogous loci through several episodes of ectopic and local duplications (Figure 3, ancientNBS-1 to -3). A segment containing two progenitor NBS sequences (ancientNBS-3 and the oppositely oriented ancientNBS-2) and the intervening CAD, duplicated ectopically, giving rise to the common ancestor of the *REN1 *locus and the chr13:11.8..12.2 Mb cluster. The third progenitor NBS sequence, that is ancientNBS-1 or one of its six locally duplicated copies, duplicated and moved ectopically to the present-day location of NBS353 (chr13:7.4 Mb), where it has remained as a dispersed gene. The founder array composed of ancientNBS-1, -2, and -3 locally proliferated through tandem duplications, involving small blocks of DNA (<10 kb) around each of the three founder genes, and generated a somewhat ordered pattern of gene distribution at the present-day chr13:21.5..21.9 Mb cluster (Figure 3 on top) [see Additional file 5].

**Figure 6:**
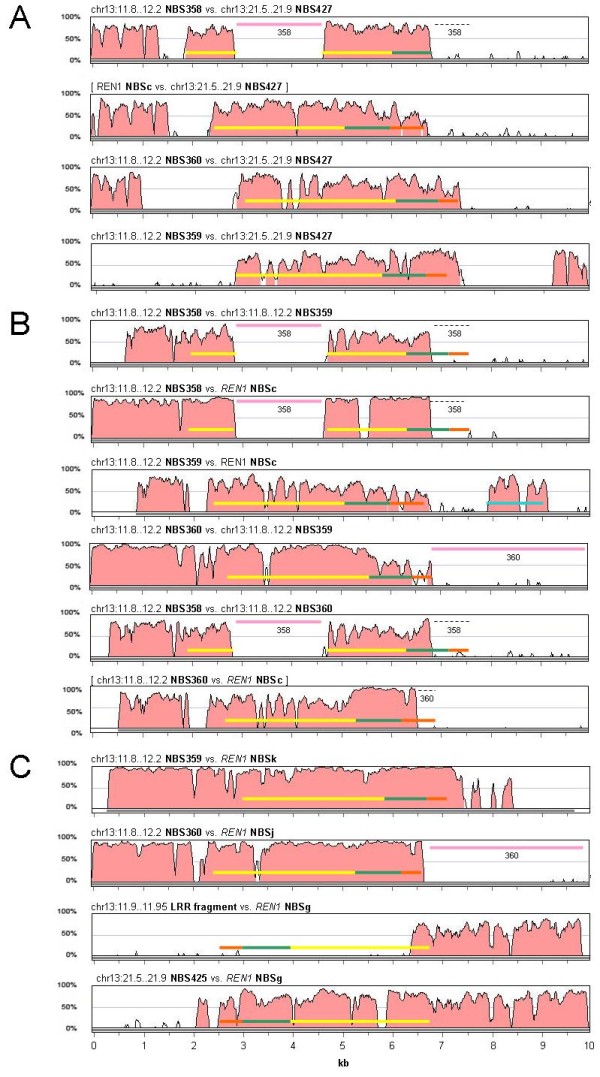
**Global alignment of 10-kb sequences around each NBS gene between paralogous clusters**. (**A**) Comparison between NBS427 in the chr13:21.5..21.9 Mb cluster and three NBS genes (NBS358, 359, 360) in the chr13:11.8..12.2 Mb cluster. Comparison with NBSc, the most closely related full length paralogue of the truncated NBS358, is included as the most reliable approximation of how NBS358 should have appeared before the Gypsy insertion in the LRR of NBS358 and the partial degeneration of the N terminus. (**B**) Comparison within chr13:11.8..12.2 Mb cluster between the three NBS genes 358, 359, and 360 (comparison with NBSc, the full length paralogue of the truncated NBS358, is included). (**C**) Comparison between each pair of the most closely related paralogous NBS genes between the chr13:11.8..12.2 Mb cluster and the *REN1 *interval. NBS425 from the chr13:21.5..21.9 Mb cluster is included in the comparison with NBSg because its paralogue in the chr13:11.8..12.2 Mb cluster has largely degenerated. The conserved blocks of nucleotide sequences and the corresponding levels of identity were calculated with LAGAN and drawn with VISTA. The coding regions corresponding to the coiled coil (orange), NBS (green), and LRR (yellow) domains are indicated by coloured bars. The NBS domain was defined as the portion comprised from the P-loop to the MHDV motif. The insertion of repetitive elements is indicated by pink bars with the identifier of the gene in which it occurred. NBSg, NBS425, and relicts of their paralogue in the chr13:11.9..11.95 Mb region are in the opposite orientation with respect to other NBS genes. DNA sequences are represented from left to right, according to the top-to-bottom orientation of the assembled genome sequence along chr13. All protein coding sequences, with the exception of NBSg, are represented in an N- to C-terminus orientation from right to left.

In the chr13:11.8..12.2 Mb cluster, NBS358 and an LRR gene remnant in the opposite orientation with respect to NBS358 are the most closely related paralogues of NBS427-428 and NBS424-426, which suggests their origin from the ancientNBS-3 and -2, respectively. NBS358 and NBS359 were derived from a common progenitor after a local tandem duplication, and diverged extensively throughout the CC, NBS, and LRR regions (the present-day NBS358 has its N terminus disrupted but its full-length paralogue in the *REN1 *locus, which is NBSc, allows an approximate prediction across the CC-NBS domains) before giving rise to NBS360. NBS360 appears to have originated from a sequence exchange which caused fusion of the LRR of NBS359 and an N-terminal region (including the coiled coil and the NBS domains) highly similar to that predicted for the intact NBS358 (Figure 6). With respect to their paralogues in the *REN1 *locus, NBS358 underwent insertion by a Gypsy transposable element in the LRR and degeneration in the N terminus, involving the 5' half of its NBS; the paralogue of NBSg was disrupted and a relict of its LRR is the only trace of its presence; and the N terminus of NBS360 was invaded by a Gypsy-like retrotransposon.

The DNA block containing an intact progenitor of NBS358, an intact paralogue descended from the oppositely oriented ancientNBS-3, a CAD, intact progenitors of NBS359 and NBS360, and distally the genes tRNA^val ^synthetase and vacuolar-sorting-protein-family-36, were the progenitor sequences that ectopically duplicated giving rise to the chr13:11.8..12.2 Mb cluster and to the segment that subsequently evolved into the *REN1 *locus.

The founder NBS genes of the *REN1 *locus were NBSc (which is paralogous to NBS358), NBSg, NBSj (which is paralogous to NBS360), and NBSk (which is paralogous to NBS359). Comparison between NBSc, NBSj, and NBSk corroborated the hypothesis that NBSj (or its paralogue NBS 360) originated by a sequence exchange, which brought together the CC-NBS domains of NBSc (or NBS358) and the LRR of NBSk (or NBS359), far beyond the boundary of the coding region on either side. The hybrid position of NBSj and NBS360 in the NJ tree of Figure 5, between the pairs NBSc/NBS358 and NBSk/NBS359, provides additional support for NBSj and NBS360 being chimeric copies generated by intergenic recombination.

Five gene islands populated by duplicated NBS and CAD sequences were distinguishable by dot plot self-comparison of the *REN1 *interval (Figure 3), [see Additional file 5]. In order to understand the relative role of single-gene tandem duplications and larger duplications in generating this expansion, the percentage of conserved sequence between each pair of 10-kb sequence around each NBS-LRR (Figure 4) was compared in the context of the percentage of conserved sequence between gene islands, which ranged in size between 118 and 186 kb. Gene islands 2-5 share 63.8 kb with 93.8% nucleotide identity, which represent 42.4% of the total length. Gene islands 3-4 share 48.5 kb (40.3% of the total length) with 94.3% nucleotide identity. Gene island 1 is more similar to 2 than to any other (90.6% identity across 64.4 kb, 39% of the total length of the pairwise comparison). The founder NBS genes are localised in three of the five gene islands (NBSc-1, NBSj-4, and NBSk-5). Gene island 2 appears to have originated from a duplication of gene island 5, which might have involved as much as 63.8 kb. Within gene island 2, NBSf has the highest similarity with NBSk, located in gene island 5, and their sequence conservation extends several kilobases outside of the coding regions (Figure 4). The other two NBS genes in gene island 2 (NBSd and NBSe) appear to have arisen in this region later by small scale duplications (see below). Gene island 3 contains NBSi, which is the most similar paralogue of NBSj, a founder NBS in gene island 4 (Figure 4). Gene island 1 is the most heterogeneous, and it has been shaped by independent small-scale duplications. NBSa is the paralogue of NBSc within the same gene island. NBSb shares the highest identity with NBSj in gene island 4, but the overlapping region does not extend significantly beyond their coding regions (Figure 4). NBSb appears to have later generated NBSe by a gene duplication, which invaded gene island 2 together with a further duplication of NBSf into NBSd. NBSg is the only founder NBS that did not undergo single gene duplication and did not remain involved in larger segmental duplications.

CAD genes in the *REN1 *interval have proliferated in a similar way [see Additional file 6]. CAD8 and CAD9 in gene island 5 are the most similar paralogues of the two CAD genes present in the chr13:11.8..12.2 Mb cluster. Within the *REN1 *interval, CAD1 and CAD2, upstream of all NBS genes, originated from a duplication of CAD8 and CAD9, after the insertion of a LINE in CAD8, in addition to the LINE shared with the progenitor CAD in the chr13:11.8..12.2 Mb cluster. CAD4 and CAD7 share high identity with CAD8, while CAD 5 and 6 show partial overlap with CAD8 in their coding sequences.

### Kinship and population analyses: genealogy of 'Kishmish vatkana' and 'Dzhandzhal kara'

**Figure 7:**
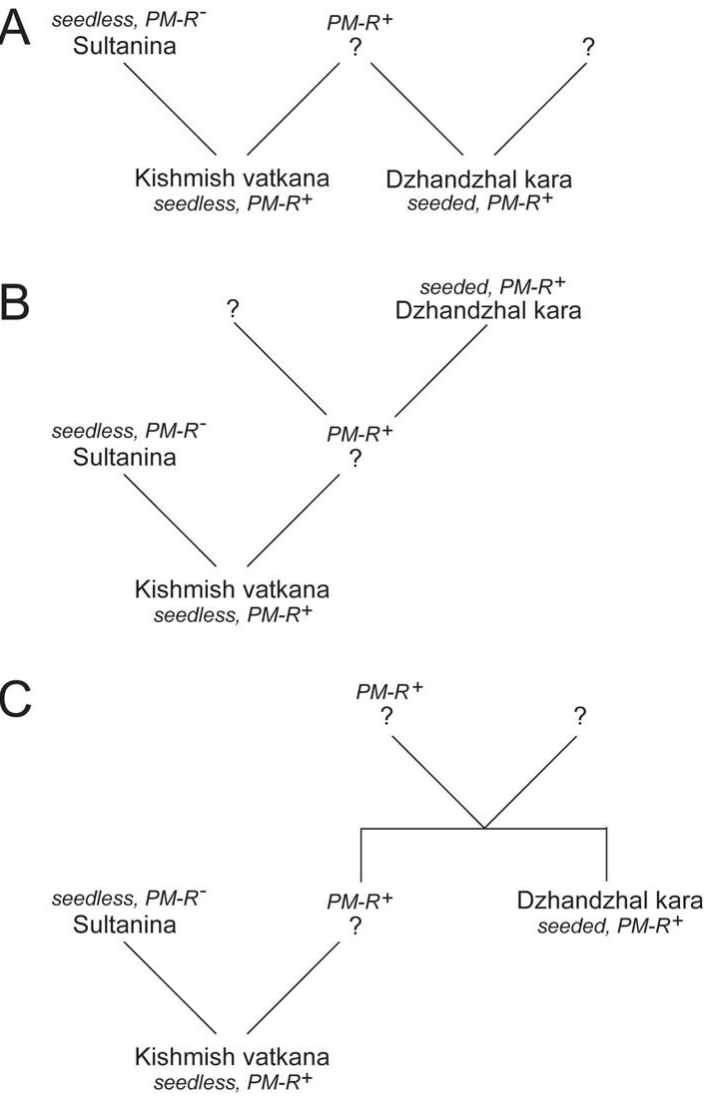
**Pedigree of 'Kishmish vatkana' and 'Dzhandzhal kara'**. A second-degree relationship was found based on 19 unlinked SSR markers, leading to the hypotheses that 'Kishmish vatkana' and 'Dzhandzhal kara' might be (**A**) half sibs of a common resistant parent, (**B**) grandparent-grandchild with 'Dzhandzhal kara' transmitting the resistance to 'Kishmish vatkana', or (**C**) avuncular.

A kinship analysis was performed using 19 unlinked SSR markers that covered all chromosomes and a small population of 16 grapevine cultivars representative of the genetic diversity in *V. vinifera *ssp. *sativa *convarietas orientalis and pontica. The sample included 'Kishmish vatkana', 'Dzhandzhal kara', several known and unknown varieties native to Central Asia, as well as some seedless cultivars that are typically grown in the same geographic region where the stenospermocarpic 'Kishmish vatkana' was originally found [see Additional file 7]. 'Kishmish vatkana' and 'Dzhandzhal kara' share at least one allele at 16 out of the 19 markers and have an estimated coefficient of relatedness (*r*) of 0.30, which is close to the expected value of *r *= 0.25 for individuals that share a second-degree relationship, that is half-siblings, grandparent-grandchild, or avuncular [39]. A likelihood ratio of 2.12 (*p *= 0.046) supported the hypothesis of a second-degree relationship versus the null hypothesis of being unrelated. 'Kishmish vatkana' and 'Sultanina' likely share a parent-offspring (PO) relationship, supported by an estimated coefficient of relatedness of 0.45. They share one allele at each of the 19 chromosome-specific loci. The likelihood ratio (LR) calculated for the PO hypothesis versus the hypothesis of being full siblings is 21.06. The PO relationship between 'Kishmish vatkana' and 'Sultanina' is supported by a high level of significance (*p *= 0.005). Following this analysis, 'Sultanina' was genotyped at 24 SSR markers evenly spaced along chr13 from VVIN62 to SC8_0149_003, including the marker SC8_0071_014, which co-segregates with *REN1*. As expected, 'Sultanina' does not have the SC8_0071_014 allele which is in coupling with *REN1 *in 'Kishmish vatkana', nor is it resistant to powdery mildew. However, 'Sultanina' does share one allele with 'Kishmish vatkana' at each locus for all markers along chr13 [see Additional file 8]. All alleles of 'Kishmish vatkana' along chr13 that are not shared with 'Sultanina' are shared with 'Dzhandzhal kara', except for SC8_0149_003 at the very bottom of the chromosome. A recombination between VVIP10 and VMC8E6 has occurred in the 'Dzhandzhal kara' chr13 tetrad of the germ cell that gave rise to the offspring 'Dzhandzhal kara' × 'Lasta'. Kinship analysis indicates that 'Sultanina' and an unknown parent carrying *REN1 *gave rise to 'Kishmish vatkana'. It is likely that the parent carrying *REN1 *is also one of the parents of 'Dzhandzhal kara', the offspring of 'Dzhandzhal kara', or a full-sibling of 'Dzhandzhal kara' (Figure 7).

**Figure 8:**
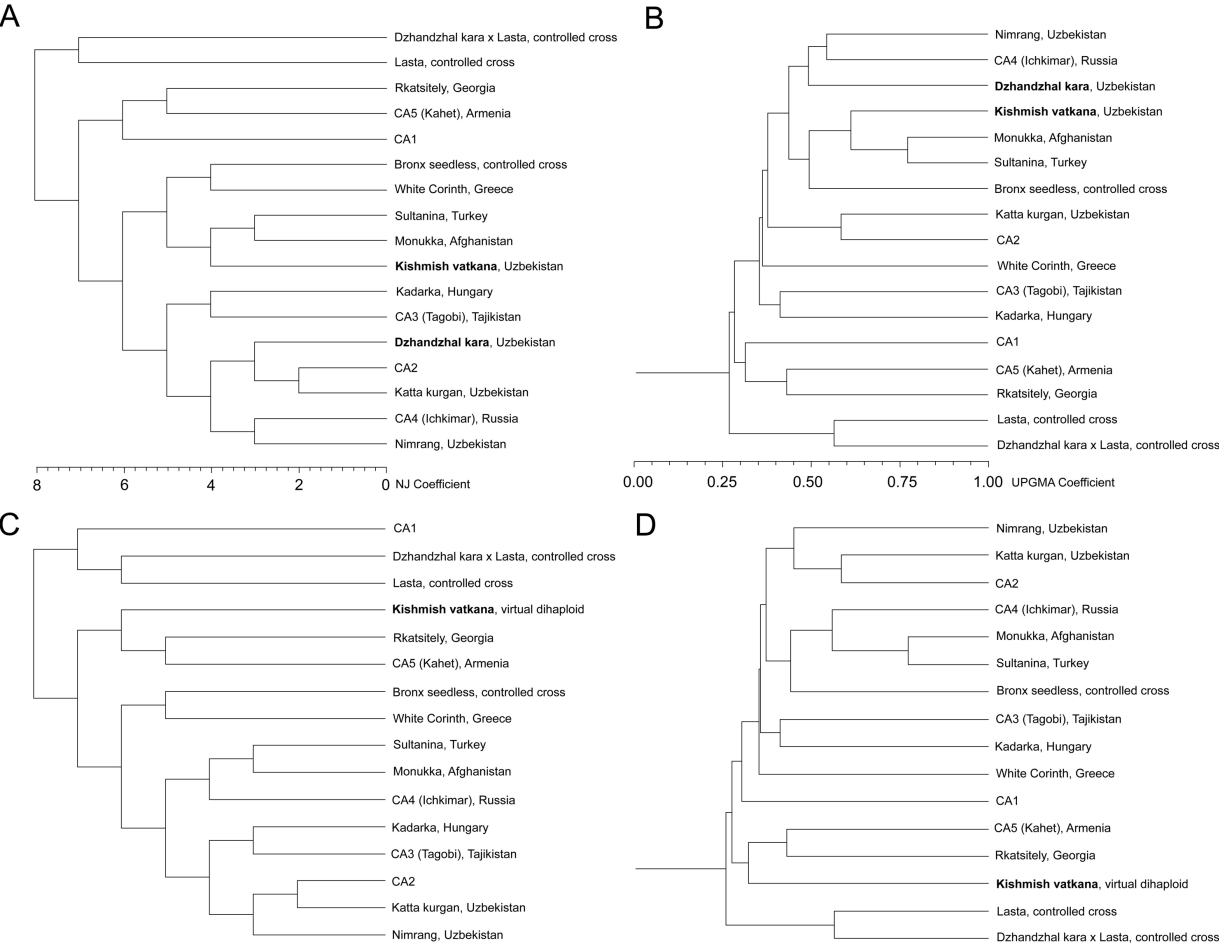
**Clustering of 'Kishmish vatkana' and 'Dzhandzhal kara' with known and unknown (CAn) varieties from Central Asia, seedless table grapes widely cultivated in Uzbekistan, Tajikistan, Turkmenistan, and Northern Afghanistan, 'Lasta', and the offspring 'Dzhandzhal kara' × 'Lasta' analysed in this paper**. The most likely identity for some unknown grapes from Central Asia is given in parentheses, and the origin of the varieties is given according to the Vitis International Variety Catalogue [40]. Genetic similarity was calculated using the Simple Match coefficient implemented in NTSYS based on 24 SSR markers covering all grape chromosomes including the 6 markers recommended by the European Vitis Database [32]. Cluster analysis was performed using the (**A**) Unweighted pair-group method with arithmetic mean (UPGMA) and (**B**) the Neighbor Joining (NJ) algorithms. Panels **C **and **D **display the clustering of the same varieties except 'Kishmish vatkana' and 'Dzhandzhal kara', which were replaced with a virtual dihaploid of 'Kishmish vatkana' constructed with the alleles not shared with 'Sultanina'.

In order to reveal more distant relatedness, not shown by the likelihood-based approach, clustering of 'Kishmish vatkana' and 'Dzhandzhal kara' with other varieties from Central Asia and seedless table grapes widely cultivated in Uzbekistan, Tajikistan, Turkmenistan, and Northern Afghanistan was performed using genotypic data at 24 loci covering all grape chromosomes and also including 6 SSR markers recommended by the European Vitis Database [32]. For Central Asian genotypes of uncertain identity not collected in official repositories, the most likely varietal assignment was attributed based on their morphological features, and the country of origin was assumed according to the Vitis International Variety Catalogue [40]. Genetic similarity was calculated using the Simple Match coefficient implemented in NTSYS, and cluster analysis was performed using the UPGMA and NJ algorithms. Neither method identified ties in the similarity matrix and each one resulted in a single tree. Both clustering methods confirmed a close relationship between 'Kishmish vatkana' and 'Sultanina', and they firmly placed 'Dzhandzhal kara' among other *V. vinifera *native to Uzbekistan and other countries in Central Asia, such as the well-known varieties 'Nimrang' and 'Katta kurgan', both of which are susceptible to powdery mildew (Figure 8).

Clustering methods applied to outcrossing diploids result in an average estimation of the two component haploid genomes present in each individual, which might obscure the contribution of outlying alleles in hybrids with a single feral parent. Utilising the parent-offspring information for 'Kishmish vatkana', the haploid genome that contributed the resistant *REN1 *allele could be separated from the haploid genome contributed by 'Sultanina'. This allowed the *in silico *construction of a di-haploid genotype which contained those alleles of 'Kishmish vatkana' that were not inherited from 'Sultanina'. The resulting virtual genotype was used for clustering with the same grape varieties as described above, in order to shed light on the phylogenetic relatedness of the resistant genotype (Figure 8). Though the virtual di-haploid did not become separated from the Central Asian group, it clustered more distantly from the Uzbek susceptible cultivars, and more tightly to grapevine varieties from Georgia and Armenia. Interestingly, this region is considered one of the centres of origin for domesticated grapes and is home to the highest genetic diversity of *V. vinifera *around the world. The gene pool analysed in this study encompassed 134 alleles at 24 loci. Only 3 alleles in 'Dzhandzhal kara' (6.2%) and 4 in 'Kishmish vatkana' (8.3%), including one allele at the *REN1*-linked locus SC8_0071_014, were not found in the reference gene pool.

In order to more widely sample the genetic variability represented in the grape germplasm, a principal component analysis was performed using the 6 markers recommended by the European Vitis Database and genotypes of 218 accessions including cultivars of *V. vinifera*, rootstocks, hybrids, and wild species [see Additional file 9]. Twenty three accessions are included in Cluster 1 [see Additional file 9], 21 of which are Western European cultivars, and two (Orion and Phönix) are interspecific hybrids with large parts of their genomes inherited from *V. vinifera *backcrosses. Cluster 2 encompasses 36 accessions: two (41B and Léon Millot) contain 50% of a non-*V. vinifera *genome, and seven are interspecific hybrids with most of the genome inherited from *V. vinifera*, while the remainder have a predominantly Western European origin. Cluster 3 is composed of Western European *V. vinifera *with a few hybrids intermixed. Cluster 4 encompasses the broadest heterogeneity, including North American species and hybrids used as rootstocks. 'Kishmish vatkana' and 'Dzhandzhal kara' grouped together in Cluster 5, along with other *V. vinifera *varieties of the proles orientalis and pontica. Cluster 6 includes only 11 varieties, including 'Katta kurgan', 'Sultanina', and 'Monukka'.

## Discussion

In this paper, we present evidence that the *REN1 *resistance allele, originally identified in 'Kishmish vatkana' [1], is also present in the genome of a second Central Asian grapevine cultivar named 'Dzhandzhal kara'. 'Kishmish vatkana' and 'Dzhandzhal kara' are estimated to share 30% of their genome by descent, in addition to sharing the resistant haplotype at the *REN1 *locus. The presence of 'Kishmish vatkana' and 'Dzhandzhal kara' was documented by Russian breeders after World War I, within a few kilometres in the Kitab-Shakhrisabz oasis, the fertile northeastern corner of the Kashkadarya River basin, south of Samarqand. 'Dzhandzhal kara' plants were also found in the district of Urgut, about 50 km to the northeast of the Kitab-Shakhrisabz oasis, on the western edges of the Pamir Mountains. While 'Kishmish vatkana' has been neglected by viticulturists and has been maintained only in germplasm collections, 'Dzhandzhal kara' has gained a wide reputation for fresh fruit production in several countries of the former Soviet Union, and it has been firmly classified as a pure *V. vinifera *in the international scientific literature.

By identifying two grapevines that share *REN1 *by descent, more information has been gained about the genealogy of these resistant grapevines. A likelihood-based kinship analysis, which predicts parent-offspring and second-degree relationships without prior knowledge of the parents [41], provided evidence that one of the parents of 'Kishmish vatkana' is 'Sultanina', also known as 'Thompson Seedless'. This parent-offspring relationship is not surprising, since both cultivars produce stenospermocarpic fruit, and 'Sultanina' has been widely cultivated for centuries in Uzbekistan and Northern Afghanistan, with the local synonym of 'Ak-Kishmish'. Since 'Kishmish vatkana' but not 'Sultanina' has a second degree relationship with 'Dzhandzhal kara', and the resistant and seedless 'Kishmish vatkana' displays a combination of biological features that are uncoupled in the parents (the pollen parent 'Sultanina' does not produce viable embryos and it is susceptible to powdery mildew, the unknown resistant parent must have been able to produce seeds), the direction of the relationship could also be established. It is not known whether the cross was made deliberately or occurred by chance in a promiscuous vineyard. However, the fact that 'Kishmish vatkana' is poorly documented in chronicles and historical records, in spite of its attractiveness as a seedless and powdery mildew resistant table grape, suggests that this hybridisation occurred rather recently.

'Dzhandzhal kara' has a longer recorded history and was already disseminated worldwide by the early 1970's, which might suggest that the seedling 'Dzhandzhal kara' is more ancient than 'Kishmish vatkana'. A grape accession catalogued as *Vitis vinifera *subsp. *vinifera *'Karadzhandal' in a US collection in 1965 is currently maintained at the USDA-ARS National Germplasm Repository in Davis, California. A variety named 'Kara dzandzal' was mentioned in a trial conducted in Japan in 1969 on 74 varieties imported from the former USSR, and recommended for breeding in a strict list of five top cultivars, including the typical Central Asian table grapes 'Katta kurgan' and 'Nimrang' [18]. A variety spelled 'Djandjal Kara' was included in a privileged set of accessions collected in Australian germplasm repositories and used in a seminal work on cultivar DNA fingerprinting in the 1990's [13].

Second-degree relationships could not be identified between 'Dzhandzhal kara' and any of the few other Central Asian grapes analysed in this paper, except for 'Kishmish vatkana'. However, the alleles carried by 'Dzhandzhal kara' at 19 SSR loci sampled over all chromosomes and the 'Kishmish vatkana' alleles at the same loci not shared with 'Sultanina' seem not to be foreign to the gene pool of *V. vinifera *ssp. *sativa *proles pontica and orientalis, which include domesticated cultivars from Central Asia and around the Caspian Sea. Thus, the kinship data, in addition to the phenotypic features of these plants, argue against close relatedness to exotic germplasm and the introduction of *REN1 *from outside of *V. vinifera*, such as East Asian genotypes or rootstocks of North American origin. Populations of *Vitis vinifera *ssp. *sylvestris*, the grapevines that climb trees in natural environments, extend far into Tajikistan and the Western Himalayas. The possibility of an infusion of genes, including resistance alleles if they exist, from indigenous and undomesticated grapevines into Central Asian varieties can not be excluded. The existence of continuous gene flow between natural and cultivated compartments of *V. vinifera *and the frequency with which these grapevines spontaneously hybridise has been recently estimated in a few Western European sites [42].

An inquiry further back into the genealogy of the powdery mildew-resistant genotypes could provide additional information regarding the origin of *REN1*. This might be accomplished by DNA analysis of more exhaustive collections of the Asian *V. vinifera *germplasm. Grape germplasm is very rich with indigenous and introduced accessions in the geographical region where the *REN1 *resistant genotypes have been found. Viticulture in fertile oases of northern Afghanistan and Uzbekistan was founded by Greek colonists in the fourth century B.C. and had already flourished by the second century B.C., at the time when General Zhang Qian brought viticulture back to China from his expeditions in the far provinces of the Western Han realm and in the modern-day Uzbekistan [43]. The geographical location of Uzbek oases on the harsh northern routes of the Silk Road accelerated the introduction of varieties traded from commercial hubs in the eastern Mediterranean basin (such as 'Sultanina', which is thought to have originated in Iran or Turkey), varieties from the Black and Caspian Seas, and grapes collected in the Xinjiang province of modern China and the Turfan depression where *V. vinifera *cultivars have been cultivated for over 2,000 years [see Additional file 10]. All of this transiting material might have mixed and cross-pollinated with locally domesticated grapes or indigenous forms of *V. vinifera *subsp. *sylvestris*. In particular, Samarqand and its surroundings, where 'Kishmish vatkana' and 'Dzhandzhal kara' were discovered, was an important hub for caravans that headed south crossing the Kashkadarya valley to Termez, the gate to northern Afghanistan, and to the junctions with the southern trade routes [see Additional file 10].

The restriction of the genetic locus in the resistant genotypes and the investigation of the genes contained in the homologous interval of the PN40024 reference sequence provided clues to the possible molecular nature of the *REN1 *gene and to the structural organisation of the locus. Functional candidates in the interval belong to two gene categories: NBS-LRR genes and cinnamyl alcohol dehydrogenases. NBS-LRRs are by far the most abundant class of plant resistance genes, in particular in the context of host-resistance and in response to biotrophs. The presence of this gene category in the *REN1 *locus is in agreement with the type of histological responses observed in inoculated leaves of 'Kishmish vatkana' [1]. The necrosis of penetrated host cells resembles the hypersensitive response (HR) triggered by a gene-for-gene type of plant-pathogen recognition. By contrast, it is not known if the presence of CAD genes in the *REN1 *locus is accidental or if it might have some biological significance. Cinnamyl alcohol dehydrogenase catalyzes the last step in the synthesis of the monomeric precursors of lignin. Different CAD classes are involved specifically in wood development or in responses to biotic stresses.

CAD inhibition partially suppresses papilla-associated cell wall appositions and jeopardises penetration resistance in powdery mildew-cereal interactions [44,45]. CADs are also involved in post-penetration resistance. Intact CAD activity is necessary for expressing an epidermal cell HR in several plant-pathogen interactions [46,47]. This observation has been explained by the role of CADs in phenylpropanoid biosynthesis products derived from cinnamyl alcohol, such as phenolic compounds and free radicals, which are integral to oxidative processes and lignin cell wall encrustations associated with HR. In grapevines, CAD transcript levels were constitutively higher in the powdery mildew resistant *V. aestivalis *'Norton' than in the susceptible 'Cabernet Sauvignon' [48]. It is noteworthy that evolution has frequently favoured physical clustering of genes belonging to widely different pathways but with related or complementary functions in defence responses (reviewed in [49]). The accumulation of favourable alleles of physically linked NBS-LRR and CAD genes that trigger and contribute to exerting HR, respectively, would provide an evolutionary advantage to the individual that inherited that block of DNA.

NBS-LRR and CAD genes and pseudogenes in the *REN1 *locus were generated through several rounds of duplication events, which appear more chaotic than duplications in the most closely related paralogous loci of the grape genome. This DNA block appears inherently prone to structural rearrangements, which makes it somewhat similar to the powdery mildew *Mla *resistance locus in barley [49], the *Rps1*-k locus in soybean [50], and *Rp1 *complex in maize [51]. In addition to segmental duplications and extensive invasion of transposable elements, NBS genes in the *REN1 *interval also evolved through intragenic recombination. The gene NBSj displayed a sharp variation of nucleotide identity immediately downstream of the MHDV site, the 3' border of the NBS domain, when compared to its paralogues NBSc and NBSk. NBSj is highly similar to the 5' end of NBSc and to the 3' end of NBSk, but substantially dissimilar to these in the complementary parts. The level of identity with either paralogue extends homogeneously beyond each end of the coding region, indicating that NBSj is more likely the result of intragenic recombination between tandemly arrayed paralogues rather than the result of domain-specific accumulation of advantageous mutations. Unequal crossing-over and gene conversion are events of nonreciprocal exchange between similar sequences, which can generate hybrid genes with novel functions by domain swapping (reviewed in [52]). Intragenic recombination frequently occurs between NBS-LRR genes and promotes diversification by reassorting domains between functional paralogues and by recruiting fragments from pseudogenes that may have accumulated mutations in the absence of selective pressure [53,54]. Either intrachromosomal gene conversions or unequal crossing-over rearranged NBS-LRR paralogues in *Arabidopsis thaliana *[53] and at the maize *Rp1 *locus [51], respectively. Both events are favoured by close physical vicinity between the partners, by high sequence similarity, and by direct orientation of the gene pair [55], all features that occur in the *REN1 *locus. In some instances, it was proven that reassortment of domains by unequal crossing over generate chimeric NBS-LRR variants associated with novel race specificities (reviewed in [51]).

The structural organisation of the 1.4-Mbp sequence across the *REN1 *locus in PN40024 bears the marks of a dynamic evolution, which suggests that this genomic region has an inherent capacity to generate high DNA variation at the population level. To pinpoint the gene that confers the *REN1 *phenotype and determine how the structure of the resistant haplotype differs across the *REN1 *locus from that in the PN40024 reference sequence, cloning and sequencing of large insert DNA BAC clones from 'Kishmish vatkana' will be initiated. The functional identification of the effective *REN1 *gene and its sequence information will shed light on the precise DNA variation that brought about the powdery mildew resistance phenotype in *V. vinifera*.

## Conclusions

We have provided evidence that the resistance gene *REN1*, which prevents grape powdery mildew, is shared by descent between two varieties of *V. vinifera*, which have been documented in Central Asia since the 1920's. In the Eurasian continent, outbreaks of powdery mildew have occurred since 1845, after the accidental introduction of the pathogen into Western Europe. The discovery of two resistant accessions that are unique in their powdery mildew resistance among the several thousands of susceptible *V. vinifera *varieties provides a model for studying the molecular evolution of a resistance gene, active against a recently introduced pathogen, in a host plant species previously thought to be entirely susceptible.

The analysis of the structural organisation of the *REN1 *locus in the reference grapevine genome sequence has provided clues to the functional categories of candidate genes present in the genetic interval, and revealed a chaotic expansion of NBS-LRR genes within the *REN1 *locus, compared to its most closely related paralogous loci in the grapevine genome. Tandemly duplicated paralogues in the *REN1 *locus and other repetitive features in the region may have created a highly unstable region prone to meiotic mispairing, which has accelerated duplications and reshuffling of domains between gene copies, as witnessed by at least one case of a chimeric NBS-LRR generated by intragenic recombination between adjacent paralogues. These events are likely to generate new functional variants, in particular when they occur within or immediately upstream of coding regions and recombine the promoter regions or functional domains originally present in separated gene copies. This scenario may have occurred in NBSj, which bears the marks of a sequence exchange that brought together the 5' promoter region, the coiled-coil domain, and the complete nucleotide-binding-site of the paralogue NBSc with the LRR region of the paralogue NBSk.

## Authors' contributions

CC performed genetic mapping and kinship analyses, and participated in drafting the manuscript; DC performed bioinformatics analyses of the structural organisation of the locus; GC assisted in the interpretation of kinship and population analyses; SH and PK carried out phenotyping of mapping populations; LK, MM, and RT assisted in the interpretation of results and contributed to the discussion. GDG conceived the design of this study, coordinated the experiments, and wrote the manuscript. All authors have read and approved the final manuscript.

## Supplementary Material

Additional file 1 SSR markers across the *REN1 *locus. Click here for file

Additional file 2 Most informative recombinant genotypes used for mapping *REN1*. Numbers across the top represent genotypes, with resistant (R) phenotypes on the left and susceptible (S) on the right. The 04-10 individuals are offspring of 'Kishmish vatkana', the 07-12 individuals are 'Dzhandzhal kara' descendents. The most informative individuals are bold faced. Markers are listed on the side according to their genetic order along chr13, with the markers that segregate with *REN1 *shown in bold. The two markers on top refer to scaffolds 73 and 175, immediately upstream of sc_47, the five markers at the bottom are placed on scaffolds 112 and 84, distal to sc_47. The resistant homologue is indicated in grey, the susceptible homologue in white. Click here for file

Additional file 3 Genes predicted in the *REN1 *interval in addition to NBS-LRRs. Click here for file

Additional file 4 NBS-LRR genes and pseudogenes predicted in the *REN1 *interval: structural features, amino acid domains, and similarity with known plant NBS-LRR resistance genes. Click here for file

Additional file 5 Dot plot comparisons between and within the *REN1 *NBS cluster and paralogous NBS clusters dispersed along the same chromosome. **(A) **Dot plot comparison between the *REN1 *interval and the chr13:11.8..12.2 Mb NBS cluster **(B) **Dot plot self-comparison of the chr13:11.8..12.2 Mb NBS cluster. **(C) **Dot plot comparison between the *REN1 *interval and the chr13:21.5..21.9 Mb NBS cluster. **(D) **Dot plot comparison between the chr13:11.8..12.2 Mb cluster and the chr13:21.5..21.9 Mb NBS cluster. **(E) **Dot plot self-comparison of the chr13:21.5..21.9 Mb NBS cluster. **(F) **Dot plot self-comparison of the *REN1 *interval. Across the panels, diagram of gene content is explained in the corresponding symbol legend. The NJ tree shows the relationship between the NBS genes. The distribution and the percentage of identity of conserved nucleotide sequences is calculated using LAGAN and drawn with VISTA. The cyan bars in (F) indicate 5 gene islands rich in NBS and CAD genes. Click here for file

Additional file 6 Global alignment of 10-kb sequences around each cinnamyl alcohol dehydrogenase (CAD) gene. (**A**) Comparison between two CAD genes in the locus chr13:11.8..12.2 Mb and CAD paralogues in the *REN1 *interval. (**B**) Comparison between the CAD founders in the *REN1 *interval (CAD8 and CAD9) and other paralogues generated by duplication. Gbrowse tracks for CAD genes in the locus chr13:11.8..12.2 Mb (top of panel A) and the CAD8 and 9 founders in the *REN1 *interval (top of panel B), displaying annotated transposable elements (TEs) and GAZE gene predictions (red/blue models). The conserved blocks of nucleotide sequences and the corresponding level of identity were calculated using LAGAN and drawn using VISTA. Click here for file

Additional file 7 (**A**) Genotypic data used for kinship analysis and for UPGMA and NJ clustering. Markers and accessions used for the calculation of relatedness and likelihood ratios are shown in bold. Markers recommended by the European Vitis Database [32] are in italics. Additional markers and accessions included in the cluster analysis conducted using NTSYS are standard font. Markers of the VChr series are described in [56], VrZag series in [57], VVMD series in [58,59], VVS series in [60], SC8_0071_014 is described in [38]. For primer sequences [see Additional file 1]. (**B**) Kinship analysis of 'Kishmish vatkana' and 'Dzhandzhal kara' with a set of 19 unlinked markers. Click here for file

Additional file 8 Haplotypes of chr13 in 'Kishmish vatkana', 'Dzhandzhal kara', and the offspring 'Dzhandzhal kara' × 'Lasta' (D × L) at 24 loci along chromosome (chr) 13. Kv chr13a, resistant homologue in 'Kishmish vatkana'; Kv chr13b, susceptible homologue in 'Kishmish vatkana', inherited from 'Sultanina'; Dzk chr13a, resistant homologue in 'Dzhandzhal kara'; Dzk chr13b, susceptible homologue in 'Dzhandzhal kara'; D × L chr13a, resistant homologue in the offspring 'Dzhandzhal kara' × 'Lasta', inherited from 'Dzhandzhal kara'; D × L chr13b, susceptible homologue in the offspring 'Dzhandzhal kara' × 'Lasta', inherited from 'Lasta'. SC8_0071_014 cosegregates with *REN1*. Click here for file

Additional file 9 Principal component analysis (PCA) via covariance matrix with data standardisation of 218 grapevine accessions including 'Kishmish vatkana' and 'Dzhandzhal kara'. Two dimensional projection along the first two coordinates on the *x *and *y *axes, explaining 25.6% and 18.3% of the variation, respectively (spreadsheet 'PCA graph'). The list of accessions attributed to the six groups identified in the graph is given in the spreadsheet 'List of accessions, PCA groups'. Click here for file

Additional file 10 Trade routes of the Silk Road, connecting the Eastern Mediterranean basin, the Middle East, Central Asia, and China (top map). The district of Samarqand, at the western foothills of the Pamir Mountains (middle map), and the sites where 'Kishmish vatkana' and 'Dzhandzhal kara' were first discovered by Russian breeders in the 1920's and 1930's (bottom map). Click here for file
