# Supplementary figures and images for: The powdery mildew resistance gene REN1 co-segregates with an NBS-LRR gene cluster in two Central Asian grapevines
Source: BMC Genet. 2009 Dec 30;10:89. doi: 10.1186/1471-2156-10-89 (PMC2814809; doi:10.1186/1471-2156-10-89)

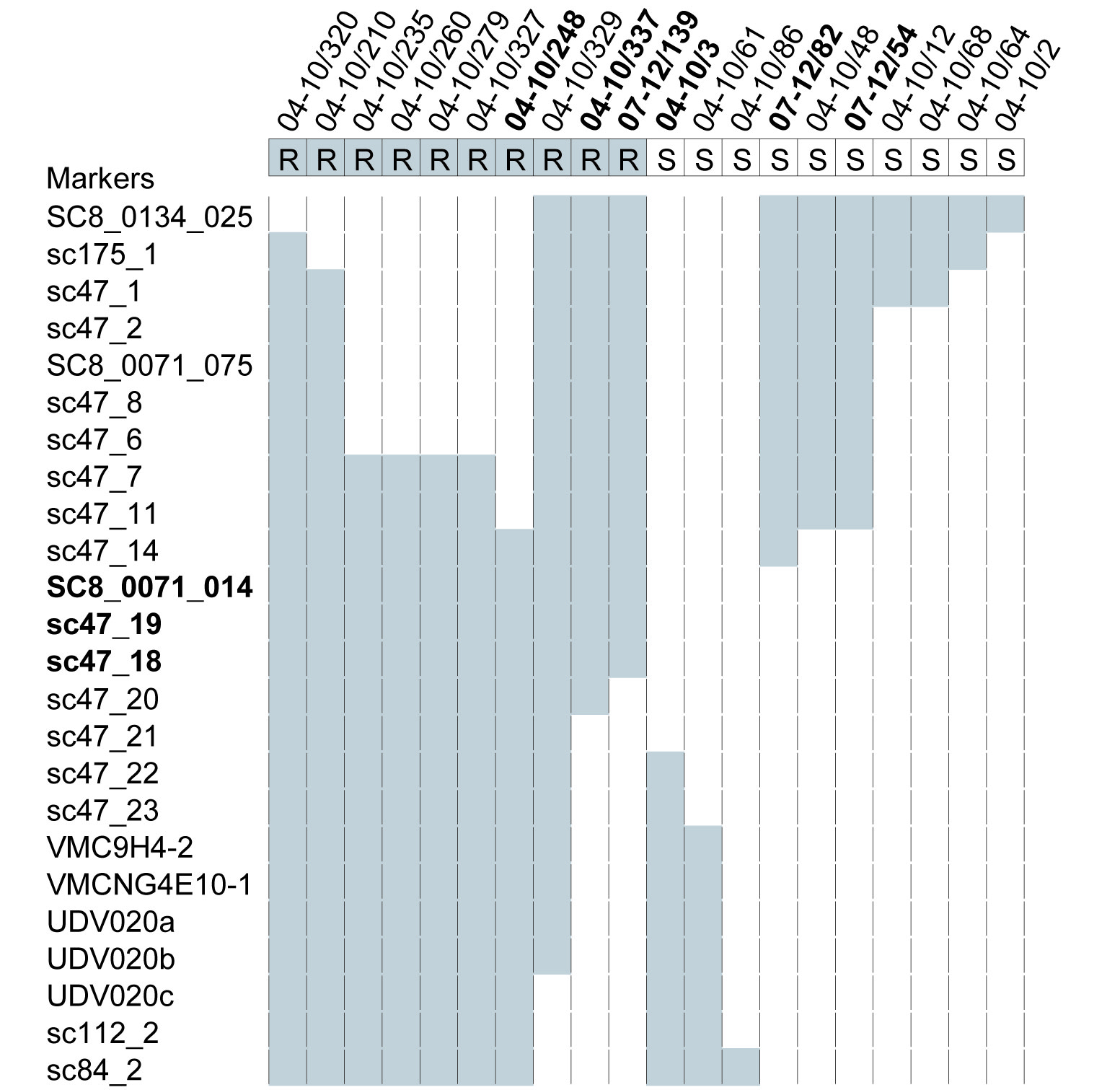

Supplement: Additional file 2 — Most informative recombinant genotypes used for mapping REN1. Numbers across the top represent genotypes, with resistant (R) phenotypes on the left and susceptible (S) on the right. The 04-10 individuals are offspring of 'Kishmish vatkana', the 07-12 individuals are 'Dzhandzhal kara' descendents. The most informative individuals are bold faced. Markers are listed on the side according to their genetic order along chr13, with the markers that segregate with REN1 shown in bold. The two markers on top refer to scaffolds 73 and 175, immediately upstream of sc_47, the five markers at the bottom are placed on scaffolds 112 and 84, distal to sc_47. The resistant homologue is indicated in grey, the susceptible homologue in white. [file 1471-2156-10-89-S2.JPEG]

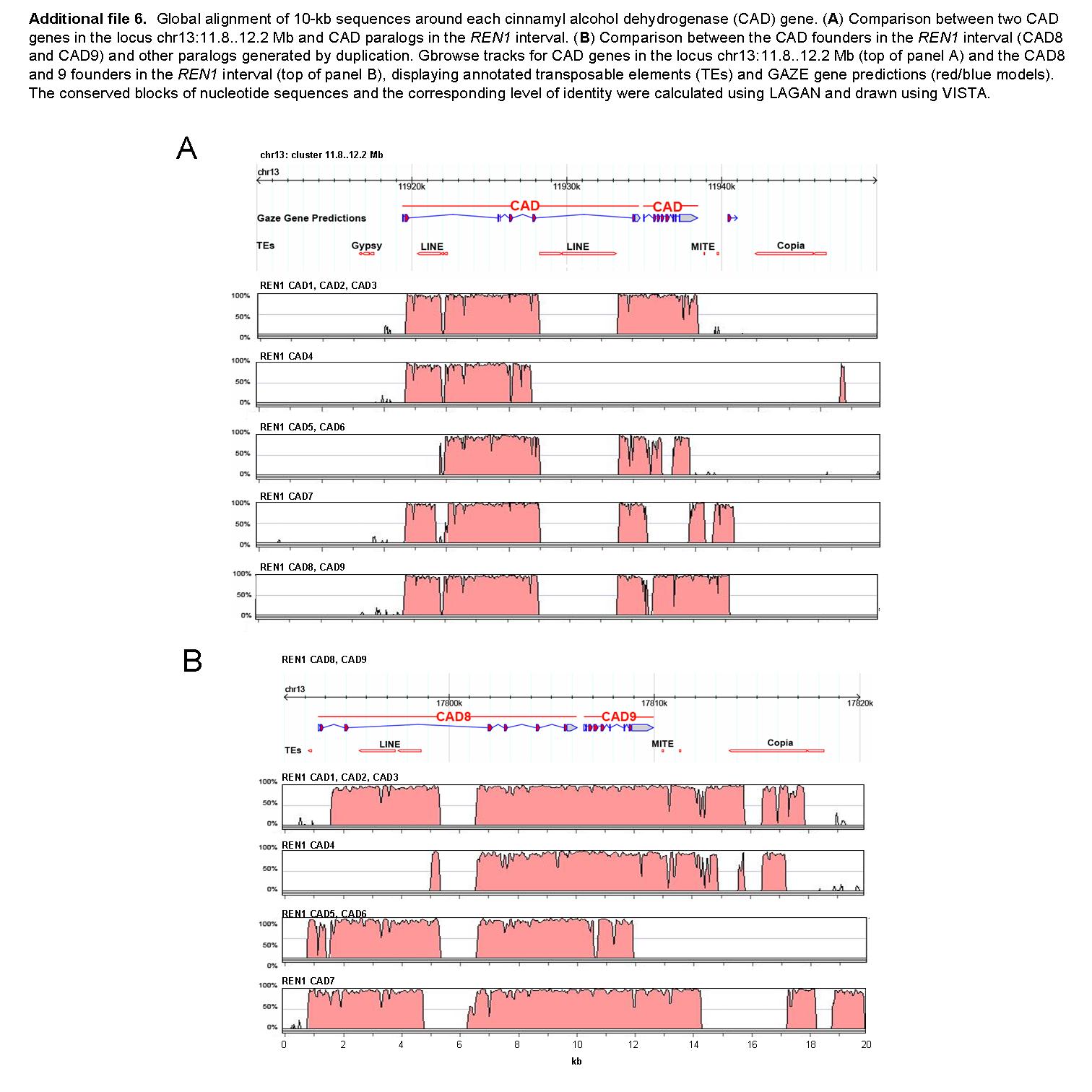

Supplement: Additional file 6 — Global alignment of 10-kb sequences around each cinnamyl alcohol dehydrogenase (CAD) gene. (A) Comparison between two CAD genes in the locus chr13:11.8..12.2 Mb and CAD paralogues in the REN1 interval. (B) Comparison between the CAD founders in the REN1 interval (CAD8 and CAD9) and other paralogues generated by duplication. Gbrowse tracks for CAD genes in the locus chr13:11.8..12.2 Mb (top of panel A) and the CAD8 and 9 founders in the REN1 interval (top of panel B), displaying annotated transposable elements (TEs) and GAZE gene predictions (red/blue models). The conserved blocks of nucleotide sequences and the corresponding level of identity were calculated using LAGAN and drawn using VISTA. [file 1471-2156-10-89-S6.JPEG]

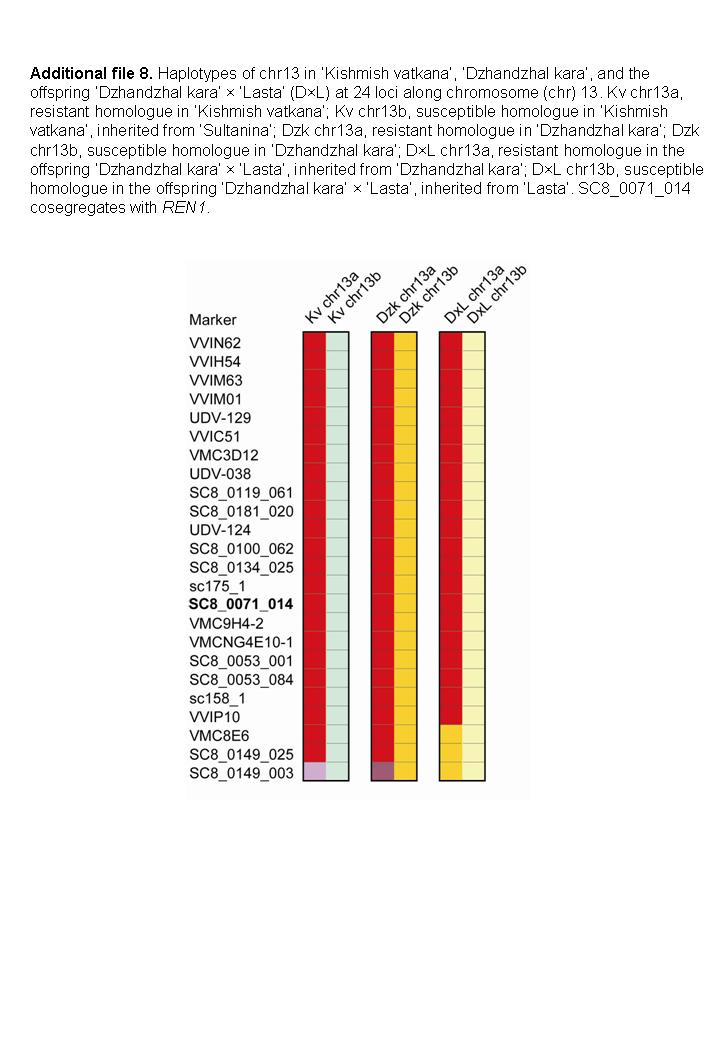

Supplement: Additional file 8 — Haplotypes of chr13 in 'Kishmish vatkana', 'Dzhandzhal kara', and the offspring 'Dzhandzhal kara' × 'Lasta' (D × L) at 24 loci along chromosome (chr) 13. Kv chr13a, resistant homologue in 'Kishmish vatkana'; Kv chr13b, susceptible homologue in 'Kishmish vatkana', inherited from 'Sultanina'; Dzk chr13a, resistant homologue in 'Dzhandzhal kara'; Dzk chr13b, susceptible homologue in 'Dzhandzhal kara'; D × L chr13a, resistant homologue in the offspring 'Dzhandzhal kara' × 'Lasta', inherited from 'Dzhandzhal kara'; D × L chr13b, susceptible homologue in the offspring 'Dzhandzhal kara' × 'Lasta', inherited from 'Lasta'. SC8_0071_014 cosegregates with REN1. [file 1471-2156-10-89-S8.JPEG]

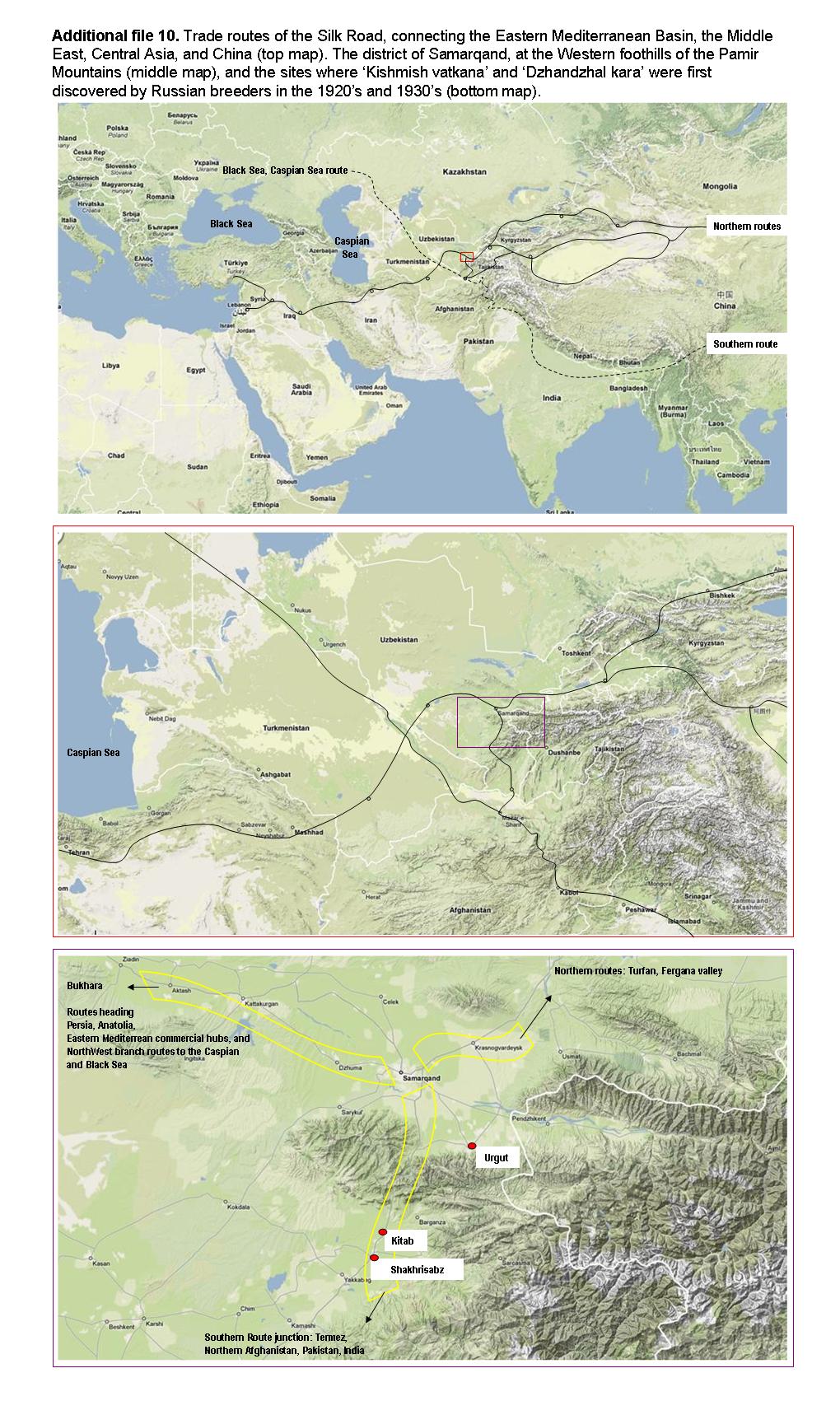

Supplement: Additional file 10 — Trade routes of the Silk Road, connecting the Eastern Mediterranean basin, the Middle East, Central Asia, and China (top map). The district of Samarqand, at the western foothills of the Pamir Mountains (middle map), and the sites where 'Kishmish vatkana' and 'Dzhandzhal kara' were first discovered by Russian breeders in the 1920's and 1930's (bottom map). [file 1471-2156-10-89-S10.JPEG]
