# Supplementary material for: The powdery mildew resistance gene REN1 co-segregates with an NBS-LRR gene cluster in two Central Asian grapevines
Source: BMC Genet. 2009 Dec 30;10:89. doi: 10.1186/1471-2156-10-89 (PMC2814809; doi:10.1186/1471-2156-10-89)

**Additional file 5. (A)** Dot plot comparison between the *REN1* interval (horizontal sequence) and the chr13:11.8..12.2 Mb NBS cluster (vertical sequence). Diagram of gene content is explained in the symbol legend. The distribution and the percentage of identity of conserved nucleotide sequences between chr13:11.8..12.2 Mb and the *REN1* locus are given on top of the *REN1* interval.

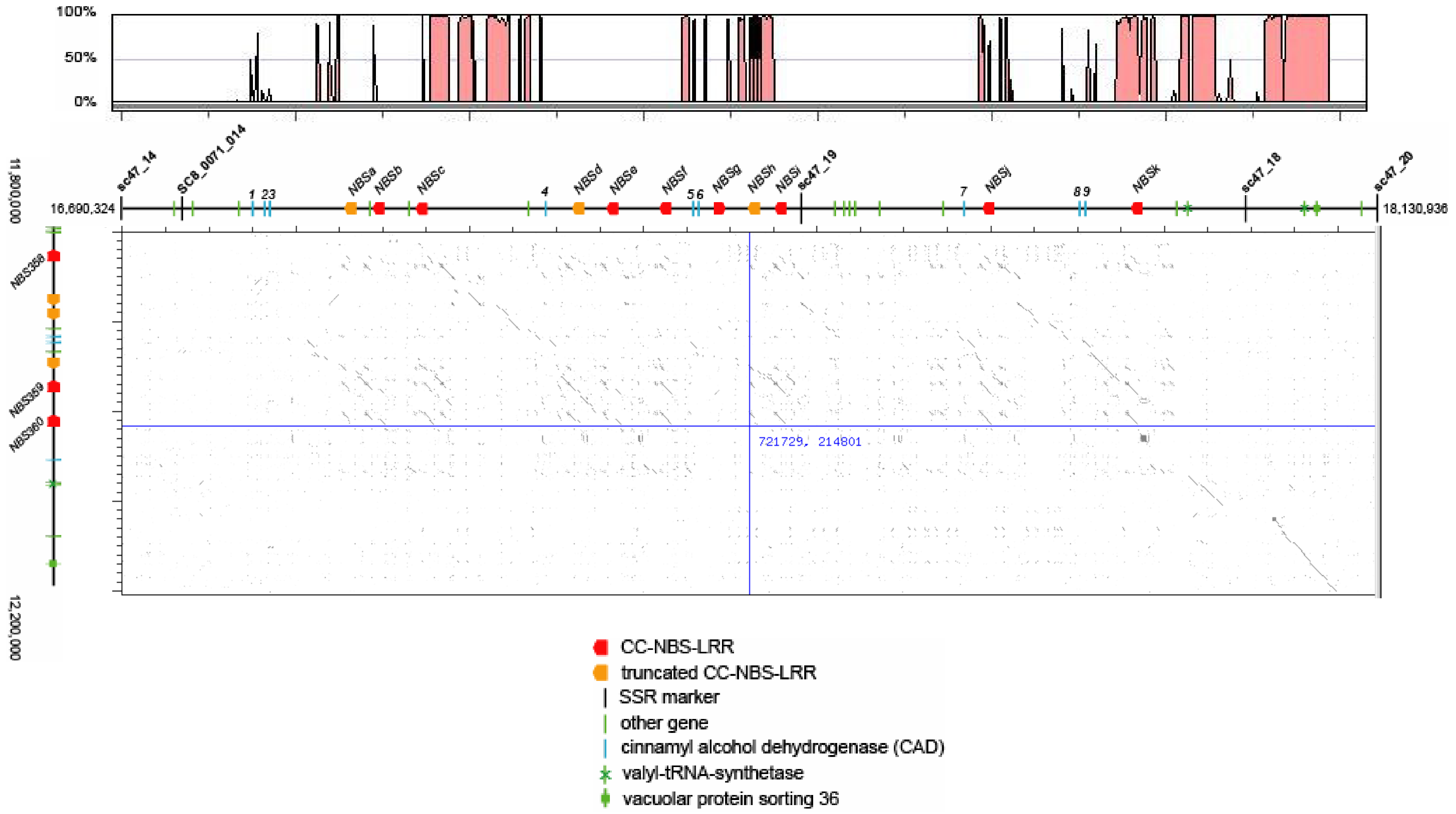

**Additional file 5. (B)** Dot plot self-comparison of the chr13:11.8..12.2 Mb NBS cluster, the phylogenetically closest paralogous locus to the *REN1* interval in the grape genome. Diagram of gene content is explained in the symbol legend. The NJ tree shows the relationship between NBS genes.

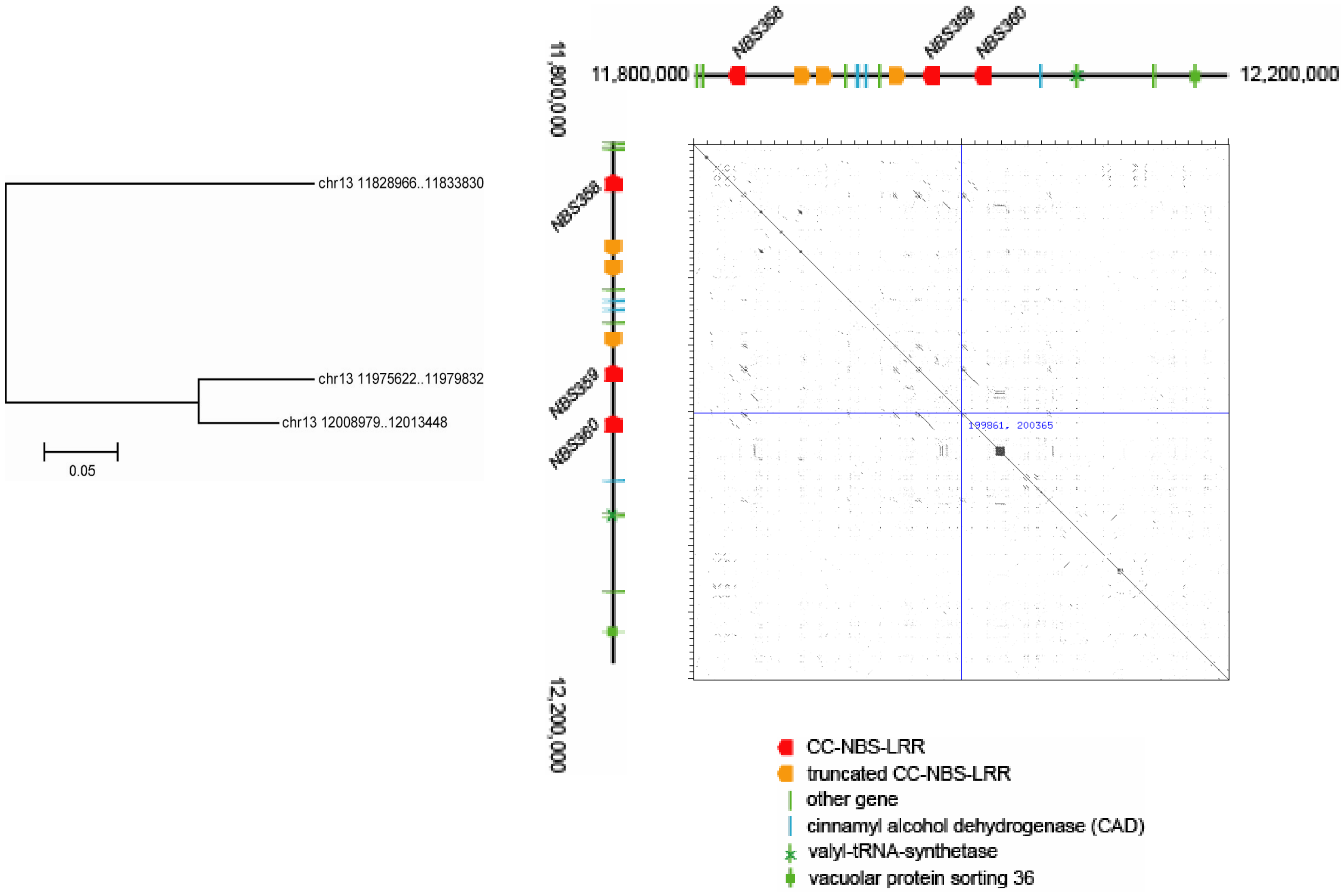

**Additional file 5. (C)** Dot plot comparison between the *REN1* interval (horizontal sequence) and the chr13:21.5..21.9 Mb NBS cluster (vertical sequence). Diagram of gene content is explained in the symbol legend. The distribution and the percentage of identity of conserved nucleotide sequences between chr13:21.5..21.8 Mb and the *REN1* locus are given on top of the *REN1* interval.

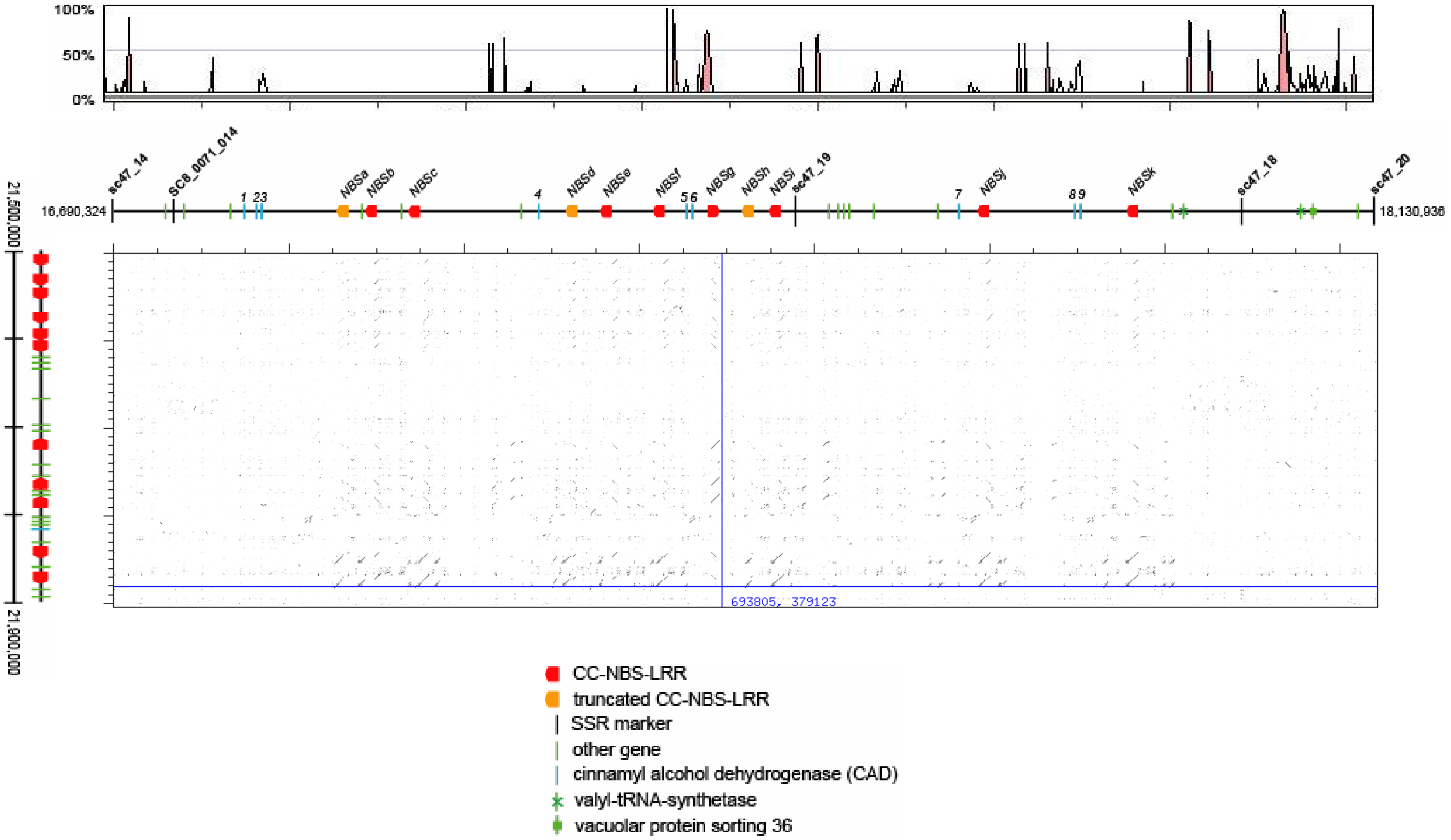

**Additional file 5. (D)** Dot plot comparison between the chr13:11.8..12.2 Mb cluster (horizontal sequence) and the chr13:21.5..21.9 Mb NBS cluster (vertical sequence). Diagram of gene content is explained in the symbol legend. The distribution and the percentage of identity of conserved nucleotide sequences are given on top and beside the corresponding sequence diagram.

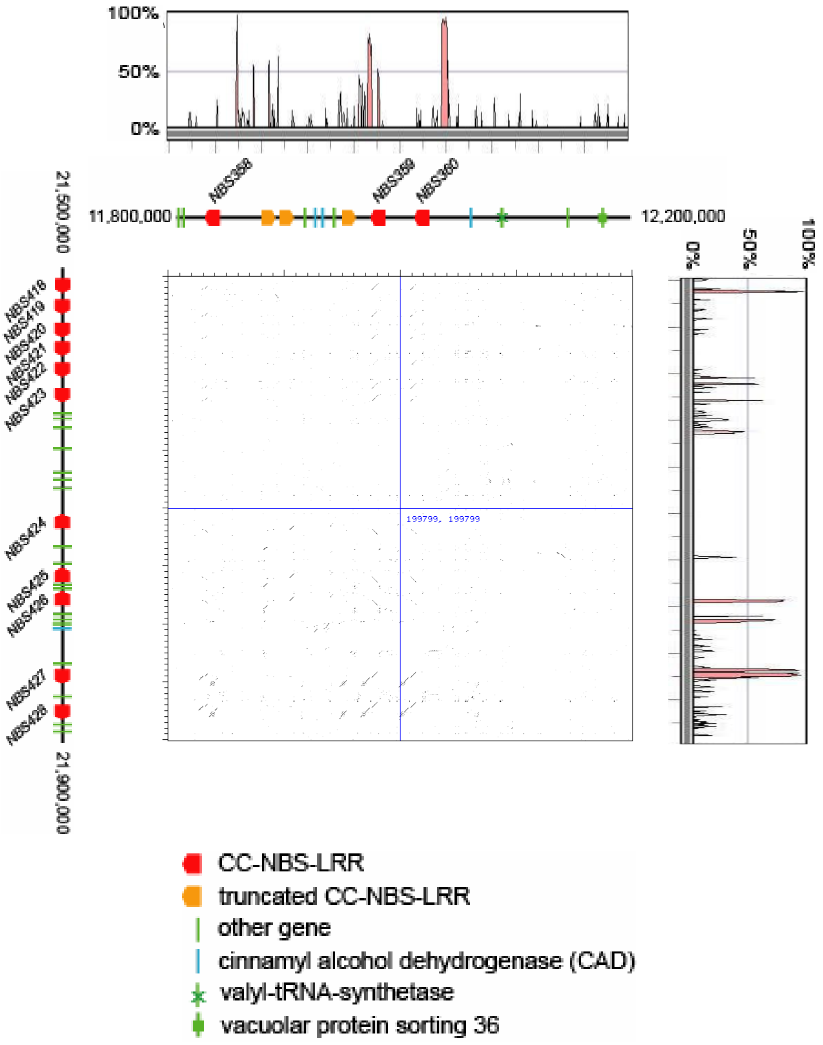

**Additional file 5. (E)** Dot plot self-comparison of the chr13:21.5..21.9 Mb NBS cluster. Diagram of gene content is explained in the symbol legend. The NJ tree shows the relationship between NBS genes.

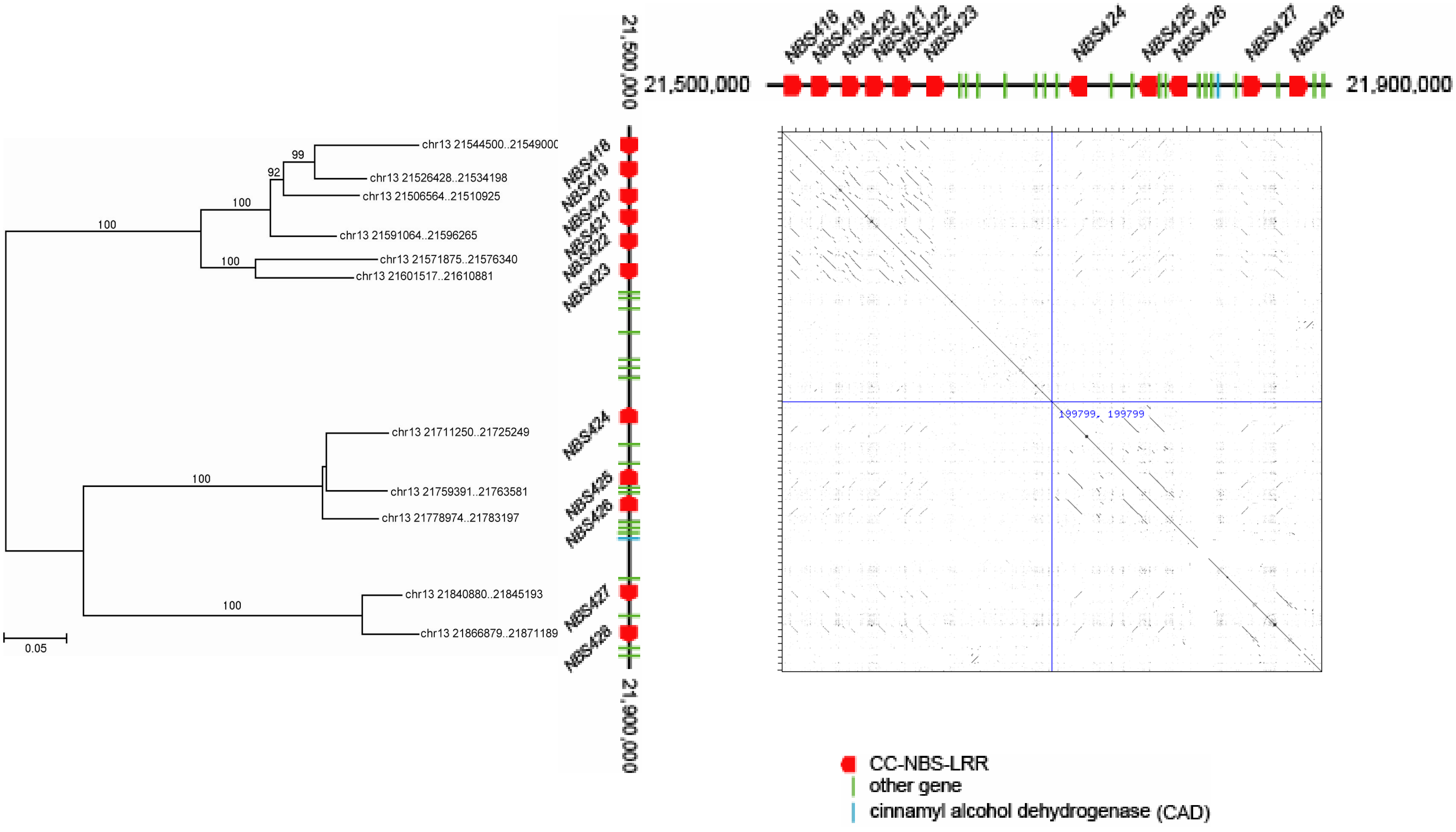

**Additional file 5. (F)** Dot plot self-comparison of the *REN1* interval. Diagram of gene content is explained in the symbol legend. The cyan bars indicate 5 gene islands rich in NBS and CAD genes.

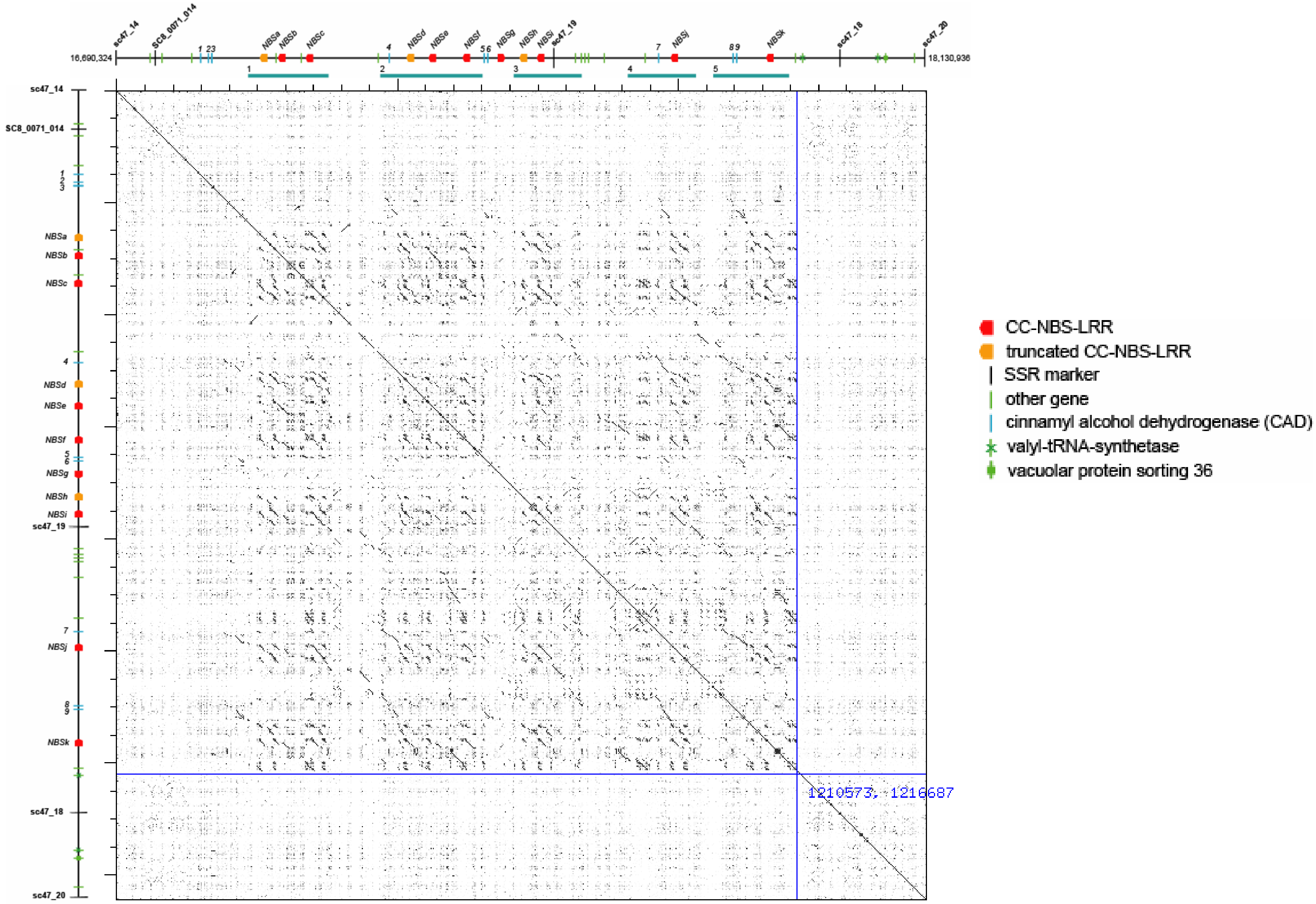

Supplement: Additional file 5 — Dot plot comparisons between and within the REN1 NBS cluster and paralogous NBS clusters dispersed along the same chromosome. (A) Dot plot comparison between the REN1 interval and the chr13:11.8..12.2 Mb NBS cluster (B) Dot plot self-comparison of the chr13:11.8..12.2 Mb NBS cluster. (C) Dot plot comparison between the REN1 interval and the chr13:21.5..21.9 Mb NBS cluster. (D) Dot plot comparison between the chr13:11.8..12.2 Mb cluster and the chr13:21.5..21.9 Mb NBS cluster. (E) Dot plot self-comparison of the chr13:21.5..21.9 Mb NBS cluster. (F) Dot plot self-comparison of the REN1 interval. Across the panels, diagram of gene content is explained in the corresponding symbol legend. The NJ tree shows the relationship between the NBS genes. The distribution and the percentage of identity of conserved nucleotide sequences is calculated using LAGAN and drawn with VISTA. The cyan bars in (F) indicate 5 gene islands rich in NBS and CAD genes. [file 1471-2156-10-89-S5.PDF]
