# Supplementary material for: The powdery mildew resistance gene REN1 co-segregates with an NBS-LRR gene cluster in two Central Asian grapevines
Source: BMC Genet. 2009 Dec 30;10:89. doi: 10.1186/1471-2156-10-89 (PMC2814809; doi:10.1186/1471-2156-10-89)
Supplement: Additional file 7 — (A) Genotypic data used for kinship analysis and for UPGMA and NJ clustering. Markers and accessions used for the calculation of relatedness and likelihood ratios are shown in bold. Markers recommended by the European Vitis Database [32] are in italics. Additional markers and accessions included in the cluster analysis conducted using NTSYS are standard font. Markers of the VChr series are described in [56], VrZag series in [57], VVMD series in [58,59], VVS series in [60], SC8_0071_014 is described in [38]. For primer sequences [see Additional file 1]. (B) Kinship analysis of 'Kishmish vatkana' and 'Dzhandzhal kara' with a set of 19 unlinked markers. [file 1471-2156-10-89-S7.DOC]

**Additional file 7.** (**A**) Genotypic data used for kinship analysis and for UPGMA and NJ clustering. Markers and accessions used for the calculation of relatedness and likelihood ratios are shown in bold. Markers recommended by the European Vitis Database [32] are in italics. Additional markers and accessions included in the cluster analysis conducted using NTSYS are regular faced. Markers of the VChr series are described in [56], VrZag series in [57], VVMD series in [58, 59], VVS series in [60], SC8_0071_014 is described in [38]. For primer sequences [see Additional file 1].

| Linkage Group | 1 | 2 | 3 | 4 | 5 | 6 | 7 | 8 | 9 | 10 | 11 | 12 | 13 | 14 | 15 | 16 | 17 | 18 | 19 | 7 | 5 | 5 | 7 | 16 |
| --- | --- | --- | --- | --- | --- | --- | --- | --- | --- | --- | --- | --- | --- | --- | --- | --- | --- | --- | --- | --- | --- | --- | --- | --- |
| Marker | **VChr1a** | **VVMD34** | **VVMD36** | **VVMD32** | ***VVMD27*** | **VVMD21** | ***VVMD7*** | **VVS4** | **VChr9a** | **VrZag64** | ***VVS2*** | **VChr12a** | **SC8_0071_014** | **VrZag112** | **VChr15a** | ***VVMD5*** | **VChr17a** | **VChr18a** | **VChr19a** | *VrZag62* | *VrZag79* | VChr5a | VVMD31 | VChr16a |
| **Nimrang** | **198:198** | **239:247** | **267:267** | **250:272** | **183:193** | **249:256** | **242:246** | **168:174** | **91:112** | **140:158** | **144:152** | **136:143** | **161:171** | **228:233** | **149:149** | **227:233** | **177:177** | **160:176** | **145:145** | 188:196 | 250:256 | 199:252 | 209:211 | 114:122 |
| **Kishmish vatkana** | **231:231** | **239:239** | **249:287** | **250:272** | **177:193** | **249:256** | **239:248** | **168:174** | **112:112** | **140:158** | **137:145** | **136:136** | **145:161** | **233:260** | **149:149** | **233:239** | **185:185** | **176:180** | **139:145** | 188:202 | 246:258 | 193:241 | 209:211 | 122:122 |
| **Katta kurgan** | **198:231** | **239:247** | **271:287** | **256:272** | **177:193** | **249:249** | **242:252** | **166:168** | **91:112** | **140:162** | **152:156** | **136:143** | **167:171** | **237:246** | **153:161** | **239:239** | **177:177** | **160:176** | **145:145** | 188:188 | 246:256 | 199:199 | 195:213 | 114:114 |
| **Lasta** | **198:223** | **239:242** | **239:295** | **256:272** | **177:187** | **256:256** | **248:250** | **167:174** | **103:112** | **143:193** | **133:150** | **136:143** | **163:165** | **233:246** | **149:153** | **237:237** | **185:185** | **152:164** | **123:137** | 186:194 | 254:259 | 183:260 | 211:213 | 114:122 |
| Dzhandzhal kara x Lasta | 198:227 | 239:242 | 269:295 | 250:256 | 177:187 | 249:256 | 242:250 | 174:174 | 103:121 | 143:193 | 150:156 | 136:143 | 145:163 | 228:246 | 149:153 | 237:239 | 177:185 | 160:164 | 123:145 | 188:194 | 246:259 | 199:260 | 195:213 | 114:122 |
| **CA2** | **198:231** | **239:239** | **267:287** | **256:258** | **177:193** | **249:249** | **233:252** | **168:174** | **91:112** | **158:162** | **152:156** | **136:143** | **171:176** | **228:237** | **146:161** | **227:239** | **185:185** | **164:176** | **145:145** | 188:196 | 246:256 | 241:241 | 211:213 | 114:114 |
| **CA1** | **210:223** | **222:239** | **263:275** | **240:258** | **179:193** | **249:256** | **246:248** | **168:174** | **91:100** | **134:193** | **135:150** | **136:136** | **161:171** | **228:247** | **142:149** | **239:239** | **177:185** | **160:164** | **133:145** | 188:196 | 248:257 | 183:194 | 211:215 | 103:110 |
| **CA4 (Ichkimar)** | **231:231** | **222:239** | **267:271** | **250:256** | **183:193** | **243:249** | **242:252** | **174:174** | **112:121** | **140:158** | **142:152** | **136:136** | **161:167** | **233:260** | **146:149** | **233:239** | **177:177** | **160:176** | **145:145** | 188:196 | 248:256 | 199:232 | 209:211 | 114:122 |
| **CA3 (Tagobi)** | **223:223** | **239:239** | **269:269** | **258:258** | **177:183** | **243:256** | **244:252** | **174:174** | **112:121** | **140:162** | **126:144** | **143:143** | **161:161** | **233:235** | **146:157** | **227:233** | **185:185** | **160:176** | **145:147** | 188:196 | 246:250 | 241:241 | 209:209 | 114:114 |
| **CA5 (Kahet)** | **223:231** | **222:239** | **253:253** | **250:272** | **177:183** | **243:256** | **239:252** | **168:172** | **115:115** | **140:158** | **135:144** | **143:143** | **147:147** | **228:233** | **142:149** | **239:239** | **177:177** | **160:168** | **139:145** | m.d. | 236:248 | 194:244 | 211:215 | 110:114 |
| **Dzhandzhal kara** | **227:231** | **239:239** | **269:287** | **250:272** | **177:193** | **249:249** | **242:248** | **174:174** | **112:121** | **143:158** | **126:156** | **136:143** | **145:176** | **228:260** | **146:149** | **233:239** | **177:177** | **160:160** | **145:145** | 188:196 | 246:246 | 183:199 | 195:209 | 114:122 |
| **Monukka** | **231:231** | **239:247** | **249:267** | **250:256** | **179:193** | **249:249** | **252:252** | **174:174** | **112:121** | **m.d.** | **m.d.** | **136:136** | **161:200** | **233:260** | **146:149** | **233:239** | **177:185** | **160:180** | **139:145** | 188:188 | 246:257 | 241:241 | 211:211 | 114:122 |
| **White Corinth** | **198:231** | **239:247** | **263:275** | **250:272** | **179:183** | **243:249** | **239:248** | **166:174** | **112:118** | **m.d.** | **133:145** | **136:143** | **164:175** | **228:233** | **149:149** | **225:239** | **177:185** | **156:176** | **139:139** | 188:200 | 242:246 | 216:241 | 205:211 | 114:114 |
| **Bronx seedless** | **198:231** | **239:247** | **249:287** | **250:272** | **179:183** | **249:249** | **239:242** | **167:174** | **105:112** | **m.d.** | **m.d.** | **136:136** | **200:200** | **241:260** | **146:149** | **233:237** | **185:185** | **160:164** | **135:139** | 188:200 | 236:246 | 241:252 | 203:211 | 103:122 |
| **Sultanina** | **231:231** | **239:247** | **249:267** | **250:250** | **179:193** | **249:256** | **239:252** | **174:174** | **112:112** | **m.d.** | **145:151** | **136:136** | **161:200** | **228:260** | **146:149** | **233:233** | **185:185** | **160:180** | **139:145** | 188:188 | 246:257 | 241:241 | 211:211 | 122:122 |
| **Kadarka** | **223:223** | **247:247** | **265:275** | **272:272** | **183:193** | **249:249** | **246:254** | **174:174** | **112:112** | **m.d.** | **m.d.** | **136:143** | **161:164** | **233:237** | **149:149** | **225:225** | **185:185** | **168:176** | **145:145** | 188:204 | 248:248 | 241:241 | 209:211 | 114:114 |
| **Rkatsitely** | **176:231** | **222:239** | **253:265** | **262:272** | **177:179** | **256:256** | **246:252** | **168:174** | **100:115** | **m.d.** | **m.d.** | **128:143** | **163:207** | **233:237** | **142:149** | **233:239** | **185:185** | **164:168** | **145:145** | 190:200 | 248:258 | 244:244 | 209:209 | 110:122 |

m.d. = missing data

**(B)** Kinship analysis of ‘Kishmish vatkana’ and ‘Dzhandzhal kara’ with a set of 19 unlinked markers

| **Pair Group** |  | ***r*** | ***p*-value for *r*** | **relationship (H')** | **null hyp (Ho)** | **LR** | ***p*-value for LR** |
| --- | --- | --- | --- | --- | --- | --- | --- |
| Nimrang | Ichkimar | 0.4869 | 0.0001 | PO | FS | 3.1101 | 0.043 |
| Kishmish vatkana | Sultanina | 0.4484 | 0.0004 | PO | FS | 21.0627 | 0.005 |
| Katta kurgan | CA2 | 0.5819 | 0 | PO | FS | 3.1101 | 0.031 |
| Bronx seedless* | Sultanina | 0.3692 | 0.0029 | PO | FS | 21.0627 | 0.003 |
|  |  |  |  |  |  |  |  |
| Monukka | Sultanina | 0.6087 | 0 | FS | HS | 13.5117 | 0.002 |
| Monukka | CA4 (Ichkimar) | 0.4709 | 0.0001 | FS | HS | 2.9061 | 0.048 |
|  |  |  |  |  |  |  |  |
| **Kishmish vatkana** | **Dzhandzhal kara** | 0.2981 | 0.0139 | HS | UR | 2.1176 | 0.046 |
| Rkatsitely | CA3 (Kahet) | 0.2291 | 0.0485 | HS | UR | 9.2788 | 0.006 |
| White Corinth | Kadarka | 0.2099 | 0.0637 | HS | UR | 2.1176 | 0.024 |
| Monukka | Bronx seedless* | 0.316 | 0.0097 | HS | UR | 2.1176 | 0.026 |
| Monukka | Kishmish vatkana | 0.356 | 0.0045 | HS | UR | 2.1176 | 0.024 |

* known pedigree: Sultanina × NY8536
